# Heterogeneous Organohydrogel Toward Automated and Interference‐Free Gradient Feeding of Drugs in Cell Screening

**DOI:** 10.1002/advs.202401720

**Published:** 2024-08-21

**Authors:** Hongxiao Gao, Xizi Wan, Wu‐Yi Xiao, Yuemeng Yang, Jingwei Lu, Shihao Wu, Li‐Ping Xu, Shutao Wang

**Affiliations:** ^1^ Beijing Key Laboratory for Bioengineering and Sensing Technology School of Chemistry and Biological Engineering University of Science and Technology Beijing Beijing 100083 P. R. China; ^2^ CAS Key Laboratory of Bio‐inspired Materials and Interfacial Science Technical Institute of Physics and Chemistry Chinese Academy of Sciences Beijing 100190 P. R. China; ^3^ University of Chinese Academy of Sciences Beijing 100049 P. R. China; ^4^ Suzhou Institute for Advanced Research University of Science and Technology of China Suzhou 215123 P. R. China

**Keywords:** automated feeding, drug screening, gradient generation, heterogeneous organohydrogel

## Abstract

Cell‐based microarrays are widely used in the fields of drug discovery and toxicology. Precise gradient generation and automated drug feeding are essential for high‐throughput screening of live cells in tiny droplets. However, most existing technologies either require sophisticated robotic equipment or cause mechanical/physiological interference with cells. Here, a heterogeneous organohydrogel is presented for automated gradient drug feeding, while ensuring minimal interference with cells. The heterogeneous organohydrogel comprises three crucial components. The bottom surface can automatically generate gradients functioning as a gradient generator, the organohydrogel bulk allows unidirectional transport of drugs without backflow, and the top surface with hydrophilic arrays can firmly anchor the cell‐based droplet array to evaluate the concentration‐dependent bioeffects of drugs accurately. Such a unique structure enables universal screening of different cell types and drugs dissolved in different solvents, requiring neither additional accessories nor arduous drug functionalization. The heterogeneous organohydrogel with unprecedented automation and non‐interference possesses the enormous potential to be a next‐generation platform for drug screening.

## Graphical abstract

A novel heterogeneous organohydrogel with automated gradient drug feeding is prepared for interference‐free cell screening. The heterogeneous organohydrogel can be applied to the screening of different cell types with minimal interference and different drugs with different solubilities, shedding new light on the design of next‐generation automated gradient feeding platforms for cell screening without additional equipment appendix.

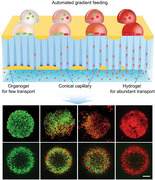

## 1 Introduction

The advancement of high‐throughput drug screening has greatly accelerated the development of modern biology,^[^ ^1^ ^]^ personalized in vitro disease models^[^ ^2^ ^]^ and toxicology studies.^[^ ^3^ ^]^ Over the past decade, considerable efforts have been devoted to the fabrication of miniaturized drug screening platforms capable of simultaneously generating concentration gradients and drug feeding. The microfluidic platform^[^ ^4^ ^]^ with delicate channel architecture allows precise control of droplet composition through the manipulation of different fluid stream splitting, mixing and reconstruction.^[^ ^5^ ^]^ It is a promising method for gradient generation^[^ ^6^ ^]^ but requires a complicated and sealed channel design,^[^ ^7^ ^]^ which significantly reduces cell accessibility for oxygen exchange and sample handling.

Recently, droplet arrays have attracted much attention due to their facile fabrication,^[^ ^8^ ^]^ high throughput,^[^ ^9^ ^]^ easy accessibility and addressability,^[^ ^10^ ^]^ enabling the parallel addition of biochemical reagents into individual cellular droplets. To obtain precise concentration gradients and multiple dosing, there are three commonly used strategies: injection, sandwich method, and stimuli‐responsive release. High‐throughput injection typically relies on sophisticated equipment to dispense liquids^[^ ^11^ ^]^ or cells into individual droplets.^[^ ^12^ ^]^ Sandwich chips provide a simple method of aligning the drug‐containing chip with the cell culture chip and then merging the two droplet arrays to facilitate precise gradient addition.^[^ ^13^ ^]^ However, this approach is limited by the loss of valuable suspension cells and the risk of cross‐contamination.^[^ ^14^ ^]^ Alternatively, various stimuli‐responsive materials have been explored to feed drugs without mechanical disruption. Micropatterned chips containing photosensitive host–guest complexes can trigger the release of drug derivatives through UV irradiation.^[^ ^15^ ^]^ The conductive heterocyclic polymer with electric‐sensitive properties was also employed to absorb or release anionic drugs.^[^ ^16^ ^]^ However, these methods need complex functionalization of drug molecules, and the introduction of additional stimulation inevitably interferes with cellular behavior.^[^ ^17^ ^]^ Ideally, a desirable drug screening system is expected to meet the following requirements: i) low sample consumption and high accessibility; ii) screening capability toward drugs in various solvents and different types of cells; iii) convenient generation of concentration gradients to evaluate cellular responses under multiple doses; iv) minimal interference with cellular behavior. It is urgent to develop a cell‐based drug screening platform that can fulfill those requirements and enable affordable drug screening for a wider range of biological laboratories.

**Figure 1:**
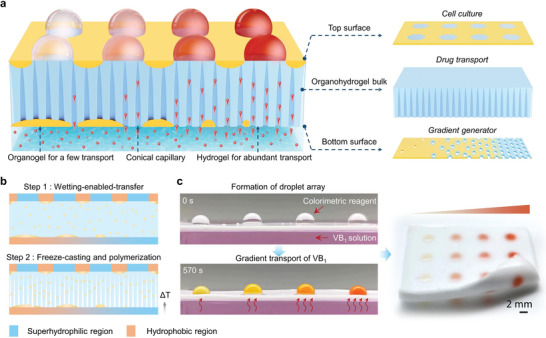
Schematic design of the heterogeneous organohydrogel. a) Schematic illustration of the heterogeneous organohydrogel with a top surface for cell culture, organohydrogel bulk for unidirectional transport and bottom surface for gradient generation. b) The preparation of the heterogeneous organohydrogel through the WET strategy and freeze‐casting process. c) Still frames taken from videos before (left‐top) and after (left‐bottom) the transport of VB_1_ molecule from the solution phase into the droplet array on the top surface. The color intensity correlates with the amount of transported VB_1_. The array shows a progressive color gradient along the direction of increasing hydrophilicity after VB_1_ feeding (right).

Herein, we prepared a heterogeneous organohydrogel with automated concentration gradient feeding for interference‐free drug screening (**Figure** **1**a) through a wetting‐enabled‐transfer (WET) strategy^[^ ^18^ ^]^ and freeze‐casting process.^[^ ^19^ ^]^ The bottom surface incorporates wettability and structural gradients for creating drug gradients, while the organohydrogel bulk contains conical capillary structures for unidirectional drug transport, synergistically resulting in automated gradient feeding. The top surface with patterned wettability was designed to capture and stabilize the cell‐based array. Quite different from the traditional feeding strategies, this platform provides an interference‐free feeding method with benefits for adherent and non‐adherent cell cultures. Along with gradient feeding of a single drug, the heterogeneous organohydrogel could be applied to multiple drugs dissolved in different solvents without any functionalization treatment. Besides, the inherent capillary force provides the primary driving force throughout the automated feeding process independent of additional energy and sophisticated equipment. Its feeding efficiency can be flexibly regulated by tuning the freeze‐casting time, the substrate wettability, and optimizing the gel monomers. We anticipate that our platform will advance existing applications in drug screening and create new opportunities in pharmaceutical studies for precision medicine and clinical applications.

## 2 Results and Discussion

### 2.1 Design of the Heterogeneous Organohydrogel

The heterogeneous organohydrogel was prepared through a synergistic strategy of WET and freeze‐casting process that involved an oil‐in‐water emulsion and two preprepared substrates with different wettability as well as a cold source (Figure 1b). Specifically, hydrogel precursor solution was composed of poly (ethylene glycol) diacrylate (PEGDA) and acrylic amide (AM) monomers, corresponding initiator and crosslinking agent. The organogel precursor composed of lauryl methacrylate (LMA) monomer, initiator, and crosslinking agent was emulsified in hydrogel precursor solution to form a stable emulsion. The as‐prepared emulsion was squeezed between two pieces of different substrates. The upper substrate is a patterned glass with superhydrophilic microwells arranged on a hydrophobic background, and the lower one is a copper substrate with a linear wettability gradient. Induced by hydrophilic/hydrophobic interactions at the solid‐liquid interface, the organogel precursor tends to be absorbed in the more hydrophobic regions of the substrate surface while the hydrogel precursor tends to be absorbed in the more hydrophilic regions, resulting in two distinctly different distributions on the two substrates synchronously. When the temperature of the copper decreases to −20 °C, a vertical temperature gradient can be generated, and ice crystals grow preferentially along the temperature gradient from bottom to top.^[^ ^20^ ^]^ After UV‐initiated polymerization (22 mW cm^−2^ of light intensity), the heterogeneous organohydrogel with a hydrophilic array on the top and a compositional gradient at the bottom was obtained. Meanwhile, by controlling the freeze‐casting time, the aligned conical capillary structures can be reserved in the organohydrogel bulk after thawing at room temperature. The hydrophilic array functions as a miniaturized cell reservoir for capturing droplets, culturing cells, and monitoring the bioeffect of drugs, the bottom surface acts as a concentration gradient generator and the organohydrogel bulk featuring conical channels provides a capillary force for efficient and unidirectional drug transport. Thus, an integrative heterogeneous organohydrogel can be obtained through WET and freeze‐casting process.

Vitamin B_1_ (VB_1_) was chosen as the model molecule to demonstrate the gradient‐feeding performance of the heterogeneous organohydrogel. As shown in Figure 1c, a droplet array containing a colorimetric reagent (0.3 mg mL^−1^ of p‐diazobenzenesulfonic acid) was formed on the top surface of the heterogeneous organohydrogel. Its bottom surface was immersed in the VB_1_ solution (labeled red for easy observation). Driven by the capillary force, the VB_1_ solution can be transported through the organohydrogel bulk and diffused into the droplet array, resulting in the generation of the red product (for details, see Experimental Section). Based on the color intensity of the droplet array, a preliminary quantification of transported VB_1_ content can be achieved. At the more hydrophobic region of the bottom surface, the droplets appear light orange‐red, indicating a few VB_1_ molecule transport and diffusion into the droplet. In comparison, at the more hydrophilic region of the bottom surface, the dark orange‐red color can be observed, which means more VB_1_ molecules have diffused into the droplet (Movie S1, Supporting Information). A gradation in color intensity was observed along the direction of increasing hydrophilicity, suggesting the successful gradient feeding of the VB_1_ molecules. The as‐prepared heterogeneous organohydrogel enables automated gradient feeding of chemicals into droplets without any other complicated sample preparation steps, which is of potential application interest in drug screening.

### 2.2 Preparation and Characterization of the Heterogeneous Organohydrogel

**Figure 2:**
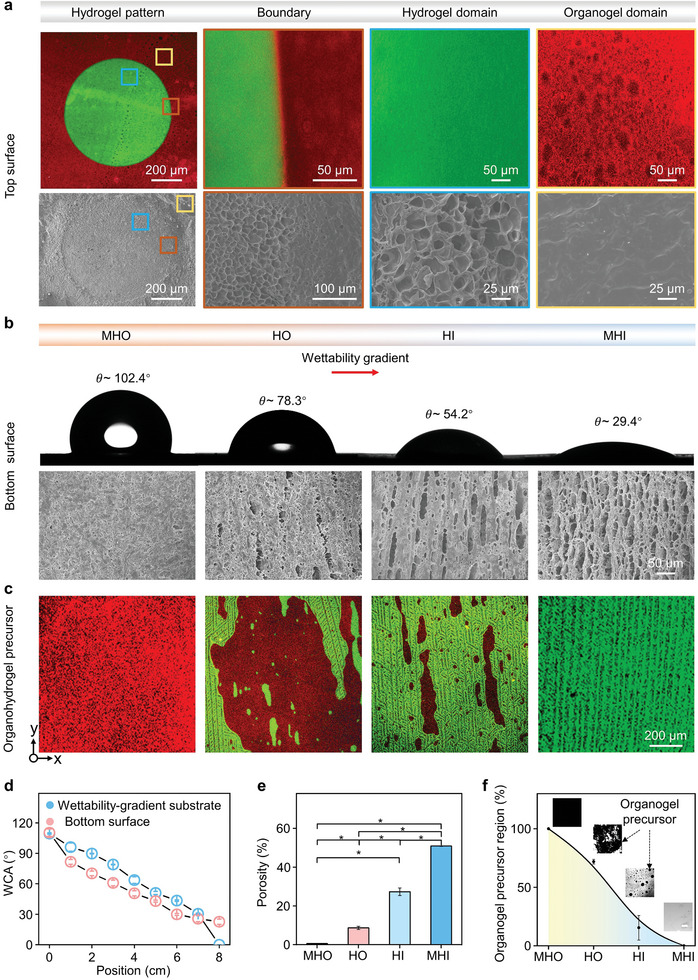
Characterization of the two surfaces of the heterogeneous organohydrogel. a) CLSM and SEM images showing the microstructure of the top surface. b) WCA and SEM images of four regions showing wettability and microstructure gradients of the bottom surface. c) The CSLM images showing the ice crystal formation of organohydrogel precursor on the wettability‐gradient substrate. When freezing on a copper with a linear wettability gradient, ice crystallization happened on the region wetted by hydrogel precursor, resulting in a gradient‐distributed ice crystal. d) WCA of the bottom surface and wettability‐gradient substrate as a function of position, respectively. e) Porosity analysis of each region on the bottom surface. *P* < 0.001 (*). f) Quantitative analysis of regions occupied by the organogel precursor on four regions of the wettability‐gradient substrate. The insets are micrographs taken by RICM showing the distribution of organogel/hydrogel precursors on the wettability‐gradient substrate. Scale bar in the inset, 200 µm. Data represent the mean ± SD (N  =  3).

Since the unique distribution of organogel and hydrogel on the top and bottom surfaces of the heterogeneous organohydrogel is critical for pinning the droplet array and creating the concentration gradients, the microstructure, chemical composition, and wettability of the two surfaces were characterized. For the top surface, by staining the hydrogel and organogel domains with hydrophilic (Rhodamine 110, green) and oleophilic (DiI, red) dyes, respectively, a circular hydrogel pattern on the organogel background can be observed from the confocal laser scanning microscopy (CLSM) images (**Figure** **2**a). A well‐defined pattern with an apparently differentiated boundary can also be observed from the scanning electron microscope (SEM) image. The hydrogel exhibited a porous network structure, while the organogel domain presented a dense and non‐porous structure. In addition, there was a distinct composition difference between the organogel and hydrogel domains. A new peak of nitrogen (N) appeared in the X‐ray photoelectron spectroscopy (XPS) spectra of the hydrogel domain, which can be attributed to the amino group in the PEGDA@AM hydrogel (Figure S1, Supporting Information). Furthermore, the organogel domain showed high hydrophobicity with a water contact angle (WCA) of ≈103° compared with the hydrogel domain with a WCA of ≈27° (Figure S2, Supporting Information). Exploiting this differential wettability and microstructure, the top surface can stabilize a robust droplet array without permeation (Figure S3, Supporting Information). Therefore, a patterned top surface has been successfully prepared and can be used for pinning a cell‐based droplet array.

The wettability and microstructure of the bottom surface were subsequently characterized. To evaluate the wettability, four regions were defined uniformly along the x‐axis direction. Their WCA showed a clear gradient, accompanied by a gradual increase in N content, indicating a compositional gradient on the bottom surface (Figure 2b; Figure S4, Supporting Information). These four regions were respectively named as more hydrophobic region (MHO) with a WCA of ≈102.4°, hydrophobic region (HO) with a WCA of ≈78.3°, hydrophilic region (HI) with a WCA of ≈54.2° and more hydrophilic region (MHI) with a WCA of ≈29.4°. Detailed analysis of the wettability along the x‐axis direction also revealed that the bottom surface exhibited a linear gradient of wettability, which was highly consistent with the wettability gradient of the substrate (Figure 2d). Meanwhile, the WCA range can be further extended from ≈0° to ≈108.2° by simply changing the monomers (Table S1, Supporting Information), which offers great flexibility to meet different screening requirements. In addition, the bottom surface exhibited a porous structure as a whole, and the porosity showed an apparent increment with increasing hydrophilicity. Quantitative analysis of the porosity on the bottom surface revealed a marked gradient increase from 0.55% to 58.88% with a decrease of WCA from ≈102.4° to ≈29.4° (Figure 2e). Thus, the wettability and structural gradients can be prepared on the bottom surface, which may allow spatial control of drug concentration for effectively regulating cell response.

To further explore the formation mechanism of the gradient‐distributed micropores on the bottom surface, we studied the evolution and crystallization of the organohydrogel precursor before polymerization on a substrate surface with a linear wettability gradient. Reflection interference contrast microscopy (RICM) with high resolution was used to observe the precise distribution of organogel/hydrogel precursors. Due to the different refractive index of the organogel and hydrogel precursors, the region occupied by the organogel precursor appears black in the RICM image, which can be distinguished from the hydrogel precursor region (Figure S5, Supporting Information). The subsequent ice crystal formation was observed under a fluorescence microscope using the setup illustrated in Figure S6 (Supporting Information). For easy distinction, the hydrogel precursor was stained green with Rhodamine 110 while the organogel precursor was stained red with DiI. As demonstrated in Figure 2f, on the more hydrophobic region of the substrate, the organogel precursor occupies 100% of this region due to hydrophobic interaction with a low underwater oil contact angle (OCA) of ≈0° (Figure S7, Supporting Information). Upon cooling, there was no ice nucleate in the region covered by the organogel precursor (Figure 2c), which can be ascribed to no phase separation that takes place during the solidification process.^[^ ^21^ ^]^ Upon polymerization, organogel with excellent hydrophobicity and a relatively dense structure on the bottom surface blocks the water pathway and inhibits the passage of drug molecules. The appearance of scattered distributed micropores on the organogel surface probably resulted from the penetration of ice crystals into the organogel precursor, thus subsequently disrupting the integrity of organogel.^[^ ^22^ ^]^ In sharp contrast, on the superhydrophilic region of the substrate, the organogel precursor occupies 0% of this region with a higher underwater OCA of ≈153°. Upon cooling, the wettability gradients induced different ice nucleation rates, which directed the preferential growth of ice crystals from the more hydrophilic region to the more hydrophobic region.^[^ ^23^ ^]^ The well‐oriented ice crystals formed by water of hydrogel precursor served as a template for the final micropore structure at the bottom surface, allowing drug molecules to pass through efficiently. Due to the presence of a linear wettability gradient on the substrate, the region occupied by organogel precursor and ice crystals varied in a gradient and therefore the micropores reserved on the obtained bottom surface after polymer infiltration showed a similar gradient. Furthermore, we calculated the spreading constant *S*  = γ_*ls*_  − (γ_*lo*_ + γ_*os*_) in different regions of wettability‐gradient substrate, where γ_*ls*_, γ_*lo*_ and γ_*os*_ are the interfacial tensions of hydrogel precursor/substrate, hydrogel precursor/organogel precursor and organogel precursor/substrate respectively. *S* > 0 indicates that the substrate surface is completely wetted by hydrogel precursor, *S* < −30 mN m^−1^ indicates that the substrate surface is completely wetted by organogel precursor, and −30 mN m^−1^ < *S* < 0 indicates that the substrate surface is partially wetted by organogel/hydrogel precursors,^[^ ^24^ ^]^ in agreement with our experimental observations (Table S2, Supporting Information). Therefore, the gradient‐distributed ice crystal can be easily achieved by the introduction of an organogel component, which is difficult to realize with pure hydrogel whether on a patterned surface^[^ ^25^ ^]^ or a wettability‐gradient surface.^[^ ^23^ ^]^

**Figure 3:**
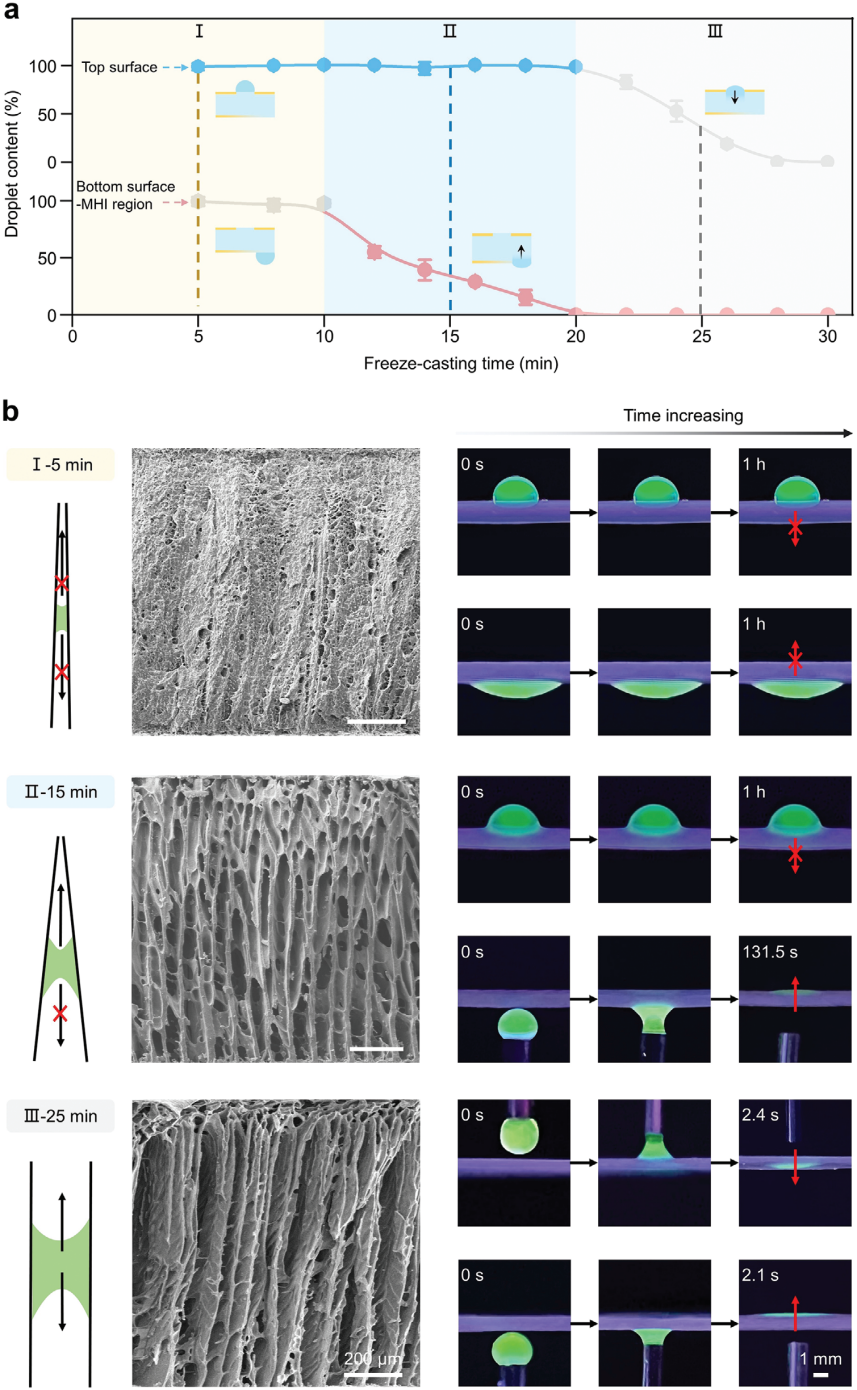
Effect of freeze‐casting time on the water transport behavior of the heterogeneous organohydrogel. a) Water content of droplets dripped on the top surface and MHI region of the bottom surface as a function of freeze‐casting time. The gray dots on the line represent droplet instability on the top surface and droplet transport failure from the bottom to the top surface, respectively. b) Cross‐sectional SEM images showing the internal structure and corresponding fluorescent images when fluorescent droplets contacted both sides of the heterogeneous organohydrogel with freeze‐casting times of 5, 15, and 25 min. Data represent the mean ± SD (N  =  3).

The freeze‐casting time plays a crucial role in regulating the capillary structure and the relevant transport behavior of the heterogeneous organohydrogel. We fabricated a series of heterogeneous organohydrogels (1 mm in thickness, 1 mm in hydrogel domain diameter) with different freeze‐casting times ranging from 5 to 30 min. A 10 µL droplet was then dripped on the top surface and MHI region of the bottom surface respectively, and the droplet content was measured. As shown in **Figure** **3**a, the droplet on the top surface remained stable even after 1 h when the freeze‐casting time was less than 20 min (blue part of the line). However, further prolonging the freeze‐casting time resulted in the penetration of the droplet array into the organohydrogel bulk, which is detrimental to the maintenance of the cellular environment. For the droplets on the bottom surface, the content of the droplet was measured after being placed for 1 min. Only when the freeze‐casting time exceeded 10 min, efficient transport into the organohydrogel bulk occurred (red part of the line). Based on the state of the droplet array on the top surface and the transport behavior on the bottom surface, the freeze‐casting time was divided into three stages. In stage I (freeze‐casting time between 5 and 10 min), the droplet array on the top surface was stabilized but the bottom surface could not be used for transport; In stage III (freeze‐casting time between 20 and 30 min), transport was achieved on the bottom surface but the droplet array was not stabilized on the top surface. Neither stage was suitable for drug screening. While in stage II (freeze‐casting time between 10 and 20 min), the obtained materials can simultaneously meet the requirements of gradient feeding through the bottom surface for drug screening and a stable droplet array on the top surface for cell culture. Therefore, such controllable transport efficiency and unidirectional transport capability were successfully realized by controlling the freeze‐casting time, ensuring the effective gradient feeding of drugs without the backflow of the cell culture medium.

To further explore the underlying mechanism for unidirectional transport of the heterogeneous organohydrogel, typical organohydrogels fabricated at different stages were selected with freeze‐casting times of 5, 15, and 25 min and their microstructure and transport behavior were compared (Figure 3b). A 10 µL droplet labeled with 1% sodium fluorescein dripped from the syringe on either the top or bottom sides of the organohydrogels to test its unidirectional transport performance. From the cross‐sectional SEM images of the heterogeneous organohydrogel and fluorescent images of water transport, it was observed that a shorter freeze‐casting time of 5 min resulted in a narrow capillary structure, which was not conducive to efficient transport from the bottom to the top surface. The fluorescent images showed that the fluorescent droplet stood firmly on both sides of the heterogeneous organohydrogel and did not penetrate even after 1 h of placement. A freeze‐casting time of 15 min resulted in an asymmetric conical capillary structure with excellent hydrophilicity (Figure S8, Supporting Information), which generated a Laplace pressure gradient to facilitate unidirectional transportation of the droplet.^[^ ^26^ ^]^ The fluorescent droplet on the bottom surface was transported completely into the organohydrogel bulk within 131.5 s while the droplet array on the top surface maintained a stable state. Further extending the freeze‐casting time to 25 min is not favorable for the stabilization of the fluorescent droplet within 3 s on the top surface due to the formation of symmetrical capillary structures. Considering the transport behavior of droplets in the MHO region of the bottom surface, the freeze‐casting time of 20 min was selected to prepare the heterogeneous organohydrogel to maximize the concentration gradient range (Figure S9, Supporting Information). In addition, increasing the thickness of the heterogeneous organohydrogel to 2 mm will increase the time to reach equilibrium and fail to generate a concentration gradient (Figure S10, Supporting Information), while decreasing the thickness to 500 µm will lead to the failure of the formation of the conical structure in the organohydrogel bulk. Therefore, a thickness of 1 mm was chosen for the preparation of the heterogeneous organohydrogel. To our knowledge, it is the first time to prepare a conical capillary structure in the organohydrogel bulk by controlling freeze‐casting time, thus facilitating automated and unidirectional feeding of drugs.

### 2.3 Gradient Generation Capacity of the Heterogeneous Organohydrogel

**Figure 4:**
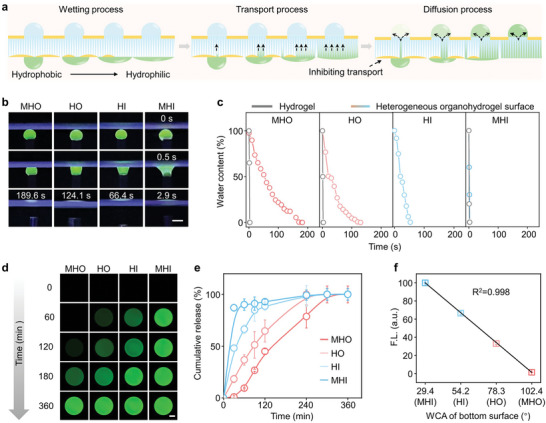
Demonstration of concentration gradient feeding property of the heterogeneous organohydrogel. a) Schematic illustration showing gradient feeding process of heterogeneous organohydrogel. The black arrows represent the direction of droplets flowing in the organohydrogel bulk and droplet array. b) Fluorescence images of the droplets (labeled with 1% sodium fluorescein) interacting with the heterogeneous organohydrogel under UV irradiation (λ = 254 nm). Scale bar: 1 mm. c) Comparison of the water droplet transport velocity of the bottom surface of heterogeneous organohydrogel and pure hydrogel, respectively. d) The fluorescence images and the corresponding release profiles e) over time showed the cumulative release of sodium fluorescein from the bottom surface to the droplet array on the top surface using the noncontact method. Scale bar: 200 µm. f) The fluorescent intensity of the droplet array and contact angles of the bottom surface is linearly related after drug transport for 20 min. Data represent the mean ± SD (N  =  3).

After optimizing the preparation conditions, the automated gradient feeding performance of the heterogeneous organohydrogel was investigated. We monitored the behavior of water droplets passing through the bottom surface into the droplet array on the top surface and three processes were considered: the wetting process, the transport process, and the diffusion process (**Figure** **4**a). During the wetting process, the droplet contacts four regions of the bottom surface with dual gradients of wettability and micropore structure. During the transport process, the droplets are driven toward the top surface by capillary force. During the diffusion process, the chemical reagents in the trailing droplets freely diffuse into the droplet arrays on the top surface. To illustrate the gradient feeding capability of the heterogeneous organohydrogel, we used the green fluorescent droplet (labeled with 1% sodium fluorescein) to mimic the drug supply and monitored its dynamic transport on the blue heterogeneous organohydrogel under UV radiation (λ = 254 nm). When the droplets contacted the different regions of the bottom surface, the droplets were transported with a gradient velocity and completely penetrated the organohydrogel bulk within 2.9, 68.4, 124.1, and 189.6 s, respectively (Figure 4b). Dynamic droplet content analysis also confirmed that the droplet can pass through the bottom surface at gradient transport velocity, which cannot be achieved with conventional hydrogel‐based transport materials^[^ ^27^ ^]^ (Figure 4c; Figure S11, Supporting Information).

Subsequently, we quantitatively characterized the dynamic diffusion of sodium fluorescein molecules into the droplet array on the top surface (1 mm in gel thickness, 1 mm in hydrogel domain diameter) by CLSM (Figure 4d). The real‐time fluorescence images of the droplet array on the top surface were recorded and the total amount of fluorescence intensity in each region was collected. As shown in Figure 4e, the cumulative release profiles of sodium fluorescein showed an initial burst release, with a plateau occurring from 20 to 300 min for each region. The relative fluorescence intensity increased linearly with the wettability of the bottom surface with a correlation coefficient of 0.998 when the drug transport time was set to 20 min (Figure 4f). Moreover, the effect of some factors including the charge and molecular weight of the drug, as well as cell density on transport efficiency were also investigated (Figure S12, Supporting Information). The gradients of doxorubicin in different solvents and at different original concentrations were also measured and recorded. As a result, we validated that the transported doxorubicin on the top surface was linearly proportional to the water contact angle of the bottom surface in different solvents and at different provided concentrations (Figure S13, Supporting Information). Therefore, once the solvent and time for drug transport were selected, we could easily modulate the target concentration by controlling the initial concentration of drugs (Table S3, Supporting Information). In addition to the concentration gradient of a single compound, muti‐component gradients can be established using the same principle. After incubation of the bottom surface in a two‐component solution (1% esculetin for green fluorescence and 0.5% doxorubicin for red fluorescence) for 20 min, a fluorescence gradient in both channels can be observed in the fluorescence images of the droplet array, confirming a gradient feeding of esculetin and doxorubicin (Figure S14, Supporting Information). Therefore, a reliable concentration gradient of drugs could be established upon a one‐step noncontact feeding, which is favorable for the practical use of the heterogeneous organohydrogel in drug screening.

### 2.4 Interference‐Free Drug Feeding Through the Heterogeneous Organohydrogel

**Figure 5:**
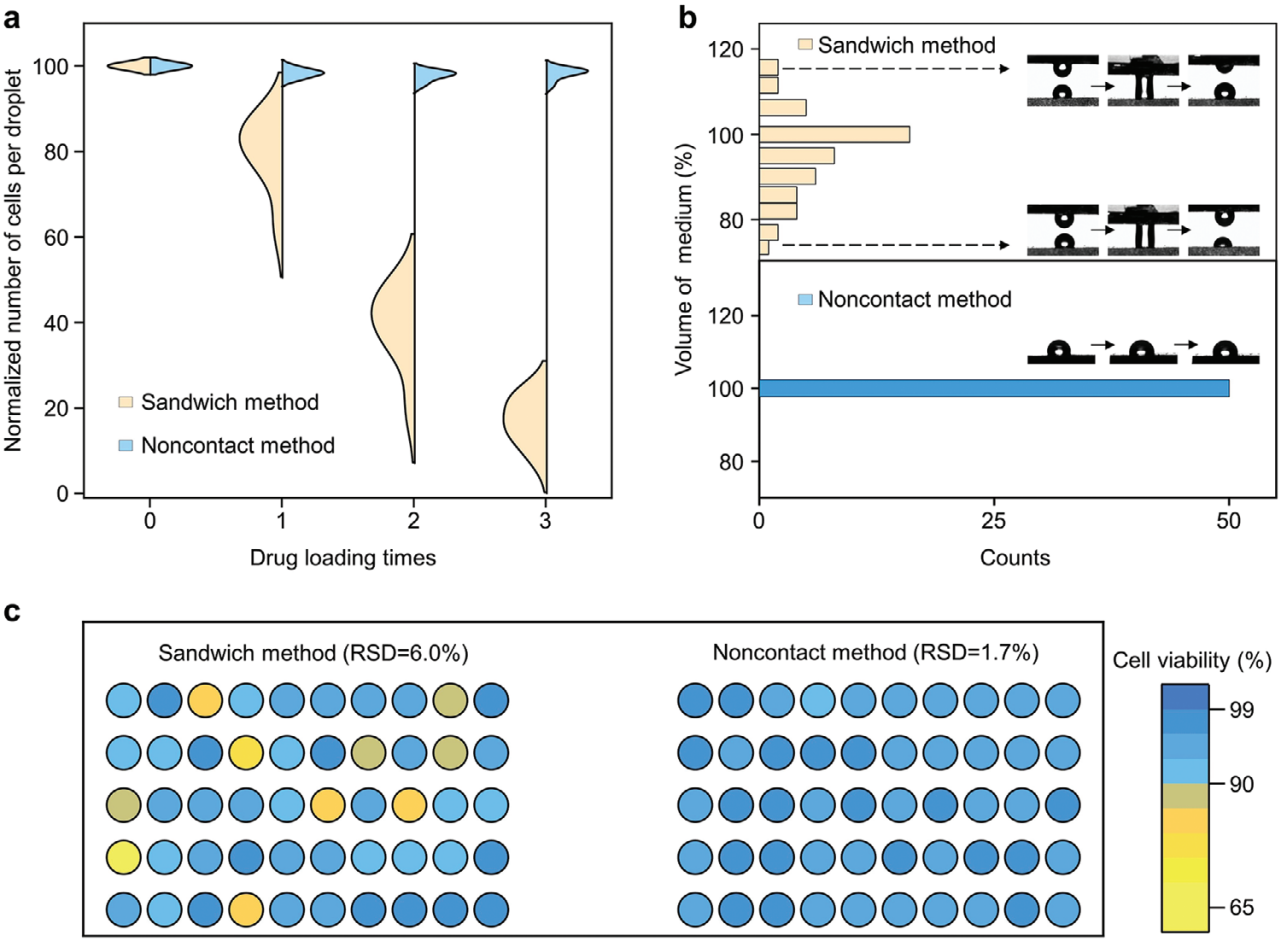
Influence of the noncontact drug feeding method on the cell‐based microarrays, taking the traditional sandwich method as a comparison. a) Quantitative analysis of the suspension cell retention through sandwich method and noncontact method based on the heterogeneous organohydrogel. b) Histograms of the culture medium volume normalized by the total amount of experiments show the residual medium volume after drug feeding. The insets are optical images indicating the volume change of the medium during drug feeding through different methods. c) The false‐color plot displayed the suspension cell viability treated with the sandwich method and noncontact method respectively.

The noncontact approach for drug feeding was compared with the traditional sandwich method. A droplet array containing 10 µL of cell suspension per microwell was formed on the top surface of the heterogeneous organohydrogel and sandwich chip respectively, followed by microscopy analysis. Alternatively, due to the difference in wettability between hydrophobic and hydrophilic domains on the top surface and the phenomenon of discontinuous dewetting, a cell‐based droplet array can also be created by simply dragging the droplet on the heterogeneous organohydrogel without manual pipetting or a liquid handling device (Figure S15, Supporting Information). For cell content, the number of cells on the sandwich chip decreased significantly with increasing dose times (**Figure** **5**a). After three doses, ≈85% of cells were lost. In comparison, the number of cells was almost unaffected by the noncontact method even after three times of drug feeding. We also monitored the change in culture medium volume after drug feeding using sandwich and noncontact methods (a 5 × 10 array), as shown in Figure 5b. The droplets processed with the sandwich method displayed inhomogeneous droplet splitting, resulting in an increased volume of some droplets and a decreased volume of others. In comparison, the culture medium in droplets remained almost unchanged after feeding by a noncontact method due to no mechanical interference. Thus, the contents of the droplet, including the cells and the culture medium, remained undisturbed on the heterogeneous organohydrogel during multiple handling. Subsequently, we explored the effect of these two feeding methods on cell viability (Figure 5c). For the sandwich method, the cells showed a lower viability with a high relative standard deviation (RSD) of 6.0% due to loss of medium limiting cell growth. In comparison, the viability of cells treated with the noncontact method was comparable to that of cells cultured on a 96‐well microplate (Figure S16, Supporting Information), with a lower RSD of 1.7%. Therefore, these results confirmed that the noncontact method is an interference‐free feeding method that benefits cell cultures, superior to conventional feeding methods via direct contact with cell‐based droplets.

### 2.5 Universality of the Heterogeneous Organohydrogel in Drug Screening

**Figure 6:**
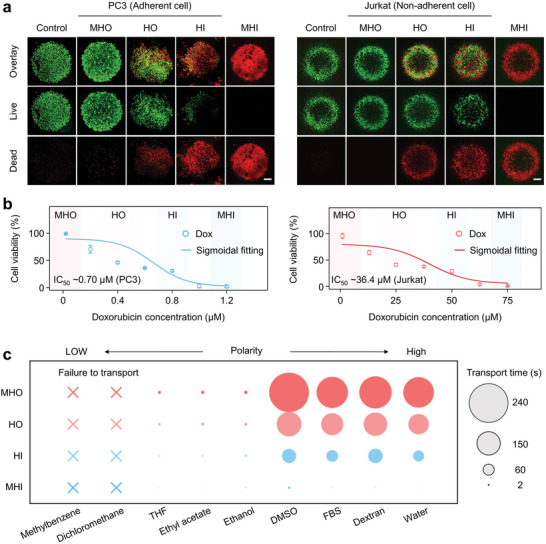
Performance and application of the heterogeneous organohydrogel in drug screening. a) Fluorescence images and b) quantitative analysis of droplet arrays containing PC3 and Jurkat cells 24 h after treatment with doxorubicin, respectively. Scale bar: 200 µm. c) The drug transport property of the heterogeneous organohydrogel in different solvents. Data represent the mean ± SD (N  =  3).

The universality of the heterogeneous organohydrogel in drug screening was investigated by studying the response of two different cell lines, including the adherent cell line (human prostate cancer, PC3) and the non‐adherent cell line (human T lymphocyte leukemia, Jurkat), to doxorubicin. To enhance cell adhesion of heterogeneous organohydrogel, a droplet containing 2.0 mg mL^−1^ (in 10 mm Tris‐HCl buffer, pH 8.5) of dopamine hydrochloride solution was dripped on the hydrogel domain for 24 h at room temperature, which results in a thin polydopamine (PDA) coating on the hydrogel domain (Figure S17a,b, Supporting Information). PC3 cells on the hydrogel domain exhibit high viability and spreading morphology. In comparison, the Jurkat cells on the top surface without PDA modification showed round contours and grew as cell clusters in suspension (Figure S17c, Supporting information). A droplet array containing 10 µL of PC3 and Jurkat cell suspensions per microwell was prepared on the top surface of the heterogeneous organohydrogel, with and without PDA modification, respectively. Then the noncontact approach was employed for one‐step drug feeding, followed by microscopy analysis. The bottom surface of the heterogeneous organohydrogel (1 mm in thickness of the heterogeneous organohydrogel, 1 mm in hydrogel domain diameter) was incubated in the doxorubicin solution (1.5 µm for PC3 cells and 75 µm for Jurkat cells) for 20 min, leading to the concentration gradient feeding of doxorubicin into the cell‐based microarray. Cell viability was then estimated 24 h after drug addition by Calcein/PI staining. Fluorescence images of PC3 and Jurkat cells indicated that more live cells were detected at the MHO region comparable to that of the control group without drug treatment, while more and more dead cells were observed with increasing hydrophilicity of the bottom surface, confirming a reliable gradient feeding of doxorubicin (**Figure** **6**a; Figure S18, Supporting Information). A clear concentration‐dependent effect of the doxorubicin on the viability of both PC3 and Jurkat cells was demonstrated in Figure 6b, and the half‐maximal inhibitory concentrations (IC_50_) of doxorubicin obtained were 0.74 µm for PC3 cells and 36.4 µm for Jurkat cells, comparable to the reported IC_50_ values (Table S4, Supporting Information). During the screening process, the heterogeneous organohydrogel floats on the culture medium, creating a high‐humidity environment. The change in droplet volume and drug concentration is less than 10% within 24 h, significantly reducing the impact of droplet evaporation on drug screening (Figure S19, Supporting Information). The gradient feeding of drugs in different solvents was also studied. For the water‐miscible solvents with different polarity (Figure 6c), including ethanol, tetrahydrofuran (THF), DMSO, ethyl acetate, fetal bovine serum (FBS) and dextran, automated gradient drug feeding can be achieved despite their differences in transport efficiency due to solubility and molecular weight. The drug‐feeding experiment was further performed using paclitaxel dissolved in DMSO and showed similar results (Figure S20, Supporting Information). Therefore, the universality of screening all cell types and various drugs with different solubilities undoubtedly endows the heterogeneous organohydrogel with tremendous potential for widespread application in drug screening.

## 3 Conclusion

In summary, we prepared a heterogeneous organohydrogel with outstanding drug‐feeding performances including automated gradient generation, efficient unidirectional transport and interference‐free cellular environment for drug screening through a WET and freeze‐casting process. By regulating the freeze‐casting time, unique conical capillary structures can be innovatively constructed in the organohydrogel bulk, enabling effective unidirectional transport. Coupled with the rational design of the wettability gradient on the bottom surface, automated gradient feeding of drugs from the solution phase to the cell‐based array on the top surface can be easily realized, addressing the general challenge of mechanical/physiological interference with cells and instrument dependency faced by traditional feeding methods. In addition, this versatile screening platform facilitates the screening of various cell types and drugs, neither requiring any drug functionalization nor being limited by solubilities. The heterogeneous organohydrogel can be prepared by a facile and scalable engineering process, holding the potential to be adopted for routine high‐throughput screening of cells and offering a promising alternative for clinical diagnostics and precision medicine.

## 4 Experimental Section

### Preparation of Superhydrophilic and Hydrophobic Substrates

The glass was cleaned with acetone and ethanol, and then rinsed with Milli‐Q three times, followed by drying with nitrogen. The glass substrate was treated with O_2_ plasma for 5 min to obtain superhydrophilicity, then put in the vacuum environment of 1H,1H,2H,2H‐perfluorodecyltrimethylsilane at 80 °C for 6 h to obtain hydrophobicity, finally treated with O_2_ plasma through the photomask (circular pattern arrays with a diameter of 2 mm and spacing of 8 mm) for 5 min to obtain superhydrophilic patterns on the hydrophobic background.

### Preparation of Copper Surface with Wettability Gradient

The copper substrate was first treated with O_2_ plasma for 5 min, and then fixed vertically on the holder with the tip facing down, and a container containing 1H,1H,2H,2H‐perfluorodecyltrimethylsilane was placed below the copper substrate. The dipped‐down speed was controlled and it was ensured that it took ≈12 min for the copper surface to be submerged. This exposed the copper substrate to the 1H,1H,2H,2H‐perfluorodecyltrimethylsilane solution for different lengths of time, generating a wettability gradient on the copper substrate along the dipping direction. The as‐prepared copper substrate was lastly rinsed with ethanol and dried under a nitrogen flow.

### Preparation of the PLMA‐PAM@PEGDA Heterogeneous Organohydrogel

First, the preparation of hydrogel precursors including 120 mg of nanoclay, 256 mg of AM, and 96 mg of DEAP were added to 2 g of water and stirred for 30 min, organogel precursors including 1 g of LMA, 39 mg of EGDMA, and 1.5 mg of DEAP were mixed and stirred for 30 min. Two grams of 15% PEGDA aqueous solution were then added into the emulsion. Subsequently, the two phases were mixed and emulsified for 1 min to form a stable oil‐in‐water emulsion. Then the emulsion was poured into the molds modified with patterned wettability and a linear gradient wettability respectively. Subsequently, the mold was placed on a refrigerator as a cooling source whose temperature was set to −20 °C, during the solidification of the solution from the cooling source, anisotropic ice crystals grew preferentially along the temperature gradient. After the sample was completely frozen, it was placed under UV light at −20 °C to initiate copolymerization for 4 h and eventually form a heterogeneous organohydrogel.

## Conflict of Interest

The authors declare no conflict of interest.

## Supporting information

Supporting Information

Supplemental Movie 1

## Data Availability

The data that support the findings of this study are available from the corresponding author upon reasonable request.
