# Supplementary material for: Heterogeneous Organohydrogel Toward Automated and Interference‐Free Gradient Feeding of Drugs in Cell Screening
Source: Adv Sci (Weinh). 2024 Aug 21;11(40):2401720. doi: 10.1002/advs.202401720 (PMC11516076; doi:10.1002/advs.202401720)
Supplement: Supplementary file 1 — Supporting Information [file ADVS-11-2401720-s002.docx]

**Supplementary information**

**Heterogeneous organohydrogel towards automated and interference-free gradient feeding of drugs in cell screening**

*Hongxiao Gao, Xizi Wan, Wu-Yi Xiao, Yuemeng Yang, Jingwei Lu, Shihao Wu, Li-Ping Xu* and Shutao Wang**

**Table of contents**

1. **Experimental Section**

1.1 Chemicals

1.2 Preparation of the heterogeneous organohydrogel

1.3 Characterization of the heterogeneous organohydrogel

1.4 The unidirectional water transport through heterogeneous organohydrogel and pure hydrogel

1.5 The effect of the noncontact method and sandwich method on droplet array

1.6 Drug treatment of PC3 and Jurkat cells on the heterogeneous organohydrogel

1.7 Statistical Analysis

**2. Supplementary Figures, Tables and Movie**

**3. Supplementary References**

1. **Experimental Section**

**1.1 Chemicals**

Inorganic nanoclay (Laponite XLS, [Mg_5.34_Li_0.66_Si_8_O_20_(OH)_4_] Na_0.66_, layer size = 20~30 nm in diameter and 1 nm in thickness, MW = 762.24.) was purchased from Rockwood Ltd, UK. lauryl methacrylate (LMA), ethyleneglycol dimethacrylate (EGDMA), acrylamide (AM), 2,2-diethoxyacetophenone (DEAP) and 1H,1H,2H,2H-perfluorodecyltrimethylsilane, doxorubicin, Polyvinyl Alcohol-1788 (PVA), Butyl Methacrylate (BMA), 3-[Dimethyl-[2-(2-methylprop-2-enoyloxy) ethyl]azaniumyl]propane-1-sulfonate (SBMA), 2-Hydroxyethyl acrylate (HEA) are bought from Aladdin Co., Ltd. (Shanghai, China). Rhodamine 110 and 1,1'-dioctadecyl-3,3,3',3'-tetramethylindocarbocyanine perchlorate (DiI) were purchased from AmyJet Scientific Co., Ltd. (Wuhan, China). The copper substrate (10 mm by 10 mm by 1 mm) was purchased from a local store. PBS buffer, DMEM medium, fetal bovine serum (FBS) and penicillin/streptomycin were purchased from Sigma-Aldrich (Germany). Deionized water (18.2 MΩ cm, Bedford, MA, USA) was purified using a Milli-Q system. All the chemical reagents were used directly without other purification.

**1.2 Preparation of the heterogeneous organohydrogel**

The PLAM/PAM@PEGDMA heterogeneous organohydrogel: The procedure was the same as described in the Experimental Section (Preparation of the heterogeneous organohydrogel).

The PLMA/PAA@PEGDMA heterogeneous organohydrogel: The procedure was the same as described in the Experimental Section (Preparation of the heterogeneous organohydrogel), except that the composition of hydrogel precursors: 1 ml of AA solution (5 mol L^-1^) and 1 ml of PVA-1788 (4 wt%) solution, 10 mg DEAP were mixed together and stirred at ambient temperature for 2 h.

The PLMA/PHEMA@PEGDMA heterogeneous organohydrogel: The procedure was the same as described in the Experimental Section (Preparation of the heterogeneous organohydrogel), except that the hydrogel precursor was changed to HEA (575 mg).

The PLMA/PSBMA@PEGDMA heterogeneous organohydrogel: The procedure was the same as described in the Experimental Section (Preparation of the heterogeneous organohydrogel), except that the hydrogel precursor was changed to SBMA (500 mg).

The PBMA/PAM@PEGDMA heterogeneous organohydrogel: The procedure was the same as described in the Experimental Section (Preparation of the heterogeneous organohydrogel), except that the organogel precursor was changed to BMA (1.42 g).

**1.3 Characterization of the heterogeneous organohydrogel**

SEM characterization: The obtained heterogeneous organohydrogel was frozen in liquid nitrogen, followed by lyophilization at -45℃ for 48 h. A filed-emission scanning electron microscope (Hitachi, SU8010) was used to characterize the structure of samples at an acceleration voltage of 10 KV. Before observation, a thin film of golden particles was coated on the heterogeneous organohydrogel to enhance electrical conductivity.

CLSM characterization: For the microstructure of the heterogeneous organohydrogel: The top surface and the bottom surface were dyed with rhodamine 110 and DiI (20 nM) for 5 min, and the excitation wavelengths were 488 nm and 549 nm respectively. The CLSM images were obtained from a laser-scanning confocal microscope (Olympus, FV1000-IX81). For the RICM: During the experiments, we raster-scanned the surface with a beam of focused monochromatic light at a wavelength of γ = 488 nm and captured the reflected light through the pinhole of a confocal microscope; thus, only the reflected light from the focal plane (i.e., the interface of interest) reaches the photomultiplier tube of the microscope. In the presence of a thin oil film, light reflected from the solid/water and water droplet/oil droplet interfaces can constructively or destructively interfere with each other. The area displaced by the organogel precursor (black area in the images) appears much darker than the area covered by the hydrogel precursor because of the smaller refractive index contrast (Δn) between glass and the organogel precursor chosen (n_glass_ = 1.52, n_organogel precursor_ = 1.455, Δn = 0.065) compared to Δn between glass and hydrogel precursor (n_hydrogel precursor_ = 1.3597, Δn = 0.1603)^1^. The relationship between reflected signal R and refractive index (n) can be expressed as:

$R= \frac{{(n1-n2)}^{2}}{\left( n1+n2 \right)^{2}}$ (1)

XPS characterization: The XPS data of the heterogeneous organohydrogel was tested on ThermoFisher Scientific ESCALAB 250Xi X-ray photoelectron spectrometer after treatment with lyophilization at -85 ℃ for 48 h.

CA measurements: An OCA-25 machine (Dataphysics Germany) was used to measure the CA of all the samples at ambient temperature. The volume of water was controlled at 5 μL.

**1.4 The unidirectional water transport through heterogeneous organohydrogel and pure hydrogel**

When observing the process of gradient transport, a syringe was placed under the heterogeneous organohydrogel or hydrogel and supplied 10 μL of fluorescence water solution (1% sodium fluorescence) continuously. The water transport performance was recorded under UV radiation (λ = 254 nm). When observing the process of fluorescent molecule diffused into the droplet array, the 10 μL of the droplet was dripped on the top surface and the bottom surface was immersed in fluorescence solution, and the fluorescence images of the droplet array were captured by CLSM. Experiment was carried out in a high-humidity and low-temperature environment to avoid evaporation.

For the VB_1_ detection:

Diazotized p-aminobenzene sulfonic acid


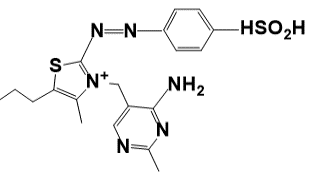

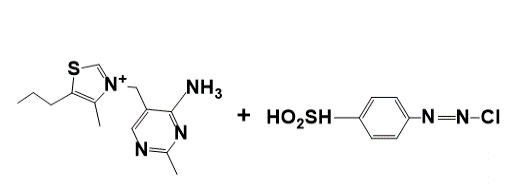


(red)

**1.5 The effect of the noncontact method and sandwich method on droplet array**

The 10 μL of a droplet containing 250 Jurkat cells labeled with cell tracker was seeding on the top surface of the heterogeneous organohydrogel and sandwich chip respectively. Cell counts and media volume were then recorded after treatment using the non-contact and sandwich methods.

**1.6 Drug treatment of PC3 and Jurkat cells on the heterogeneous organohydrogel** Heterogeneous organohydrogel was prepared as described above. To enhance cell affinity and favored cell adhesion of PC3 cells, the polydopamine coating was deposited on the hydrogel domain by dripping the 2 mg∙mL^−1^ dopamine hydrochloride solution in 10 mM Tris-HCl buffer at pH 8.5 for 24 h. Then the heterogeneous organohydrogel was immersed in water for 7 days to remove unreacted monomers and reagents.

For seeding cells on the heterogeneous organohydrogel, cells were trypsinized and diluted with DMEM containing 15% FBS and 1% penicillin/streptomycin till concentrations of 3.0 × 10^5^ and 1.2 × 10^5^ cells mL^−1^ for PC3 and Jurkat seeding, respectively. 10 μL of cell suspension was dispensed onto the top surface and incubated 24 h before drug treatment in a cell incubator containing 5% CO_2_.

For drug screening experiments of doxorubicin, the bottom surface of the heterogeneous organohydrogel was floated on a concentration of doxorubicin (75 μM for PC3 and 1.5 μM for Jurkat respectively) and incubated for 20 min. After drug treatment, a cells-based droplet array on the heterogeneous organohydrogel surface was incubated in a DEME medium for 24 h. Afterward, cells were stained with Calcein and PI (1.5 ug/ml) for 10 min through a noncontact method. For drug screening experiments of the paclitaxel, the bottom surface of the heterogeneous organohydrogel was floated on paclitaxel solution (350 μM in DMSO) and incubated for 30 min. Dose-response curves were plotted in Origin using the “nonlinear curve fit” function: category “Growth/Sigmoidal,” function “DoseResp,” IC50 values were calculated in Origin after curve fitting.

**1.7 Statistical Analysis**

Error bars on all published graphs represent standard deviations. All experiments were repeated 3 times, and data from one representative experiment is presented.

**The capillary rise dynamics analysis of droplets in the process of water transport through the bottom surface of the heterogeneous organohydrogel**

The heterogenous organohydrogel is a typical porous media in which the height $h$ reached by the liquid front is given by the Washburn equation^2^:

$h^{2}=\frac{r\gamma_{L}\cos\theta}{2\eta}t$ (S3)

Where $r$ is the mean static radius of the capillary, $\eta$ is the viscosity of the liquid, $\gamma_{L}$is the surface tension of the liquid, and $\rho$ is the liquid density.

The weight $w_{T}$ of the droplet through the hydrophilic region is related to the height in the cylinder by

$w_{T}=\epsilon\rho\pi R^{2}h$ (S4)

Where $\epsilon$ is the porosity of the bottom surface of the heterogeneous organohydrogel, $\rho$ is the density of the liquid, and R is the inner radius of the capillary structure.

Finally, after combining Eq. (S4) with Eq. (S3), we can obtain

${w_{T}}^{2}=(r\epsilon^{2}{(\pi R^{2})}^{2})\frac{\rho^{2}\gamma_{L}\cos\theta}{2\eta}t$ (S5)

For the hydrophobic region of the bottom surface of the heterogeneous organohydrogel, hydrophilic capillaries in the bulk and hydrophobic surface microwell form a Janus structure, and the driving force from the capillary force on the water droplet ($F_{ca}$) can be calculated as follows^3,4^:

$F_{ca}=2\pi\eta r(\cos\theta_{2}-\cos\theta_{1})$ (S6)

Where $\cos\theta_{2}$ and $\cos\theta_{1}$ are the water contact angle of the internal capillary channel in the organohydrogel bulk and hydrophobic region of the bottom surface. The weight $w_{T}$ of droplets transported through hydrophobic regions can be calculated as:

${w_{T}}^{2}=(r\epsilon^{2}{(\pi R^{2})}^{2})\frac{\rho^{2}\gamma_{L}(\cos\theta_{2}-\cos\theta_{1})}{2\eta}t$ (S7)

And the residual water content $w_{R}$% can be calculated by：

$w_{R}\%=\frac{w_{total}-w_{T}}{w_{total}}$ (S8)

Where $w_{total}$is the original weight of the droplet before transport. For the other regions such as HI and HO with both hydrophobic and hydrophilic domains, Eq. (S6) and Eq. (S7) can be combined according to the ratio of hydrophobic and hydrophilic regions. As shown in Figure S11, the theoretical residual water content ($w_{R-theo.}$) is consistent with the experimental data ($w_{R-Exp.}$). Therefore, the gradient feeding performance of the heterogeneous organohydrogel is induced by the synergistic effect between wettability and structure.

**2. Supplementary Figures, Tables and Movie**


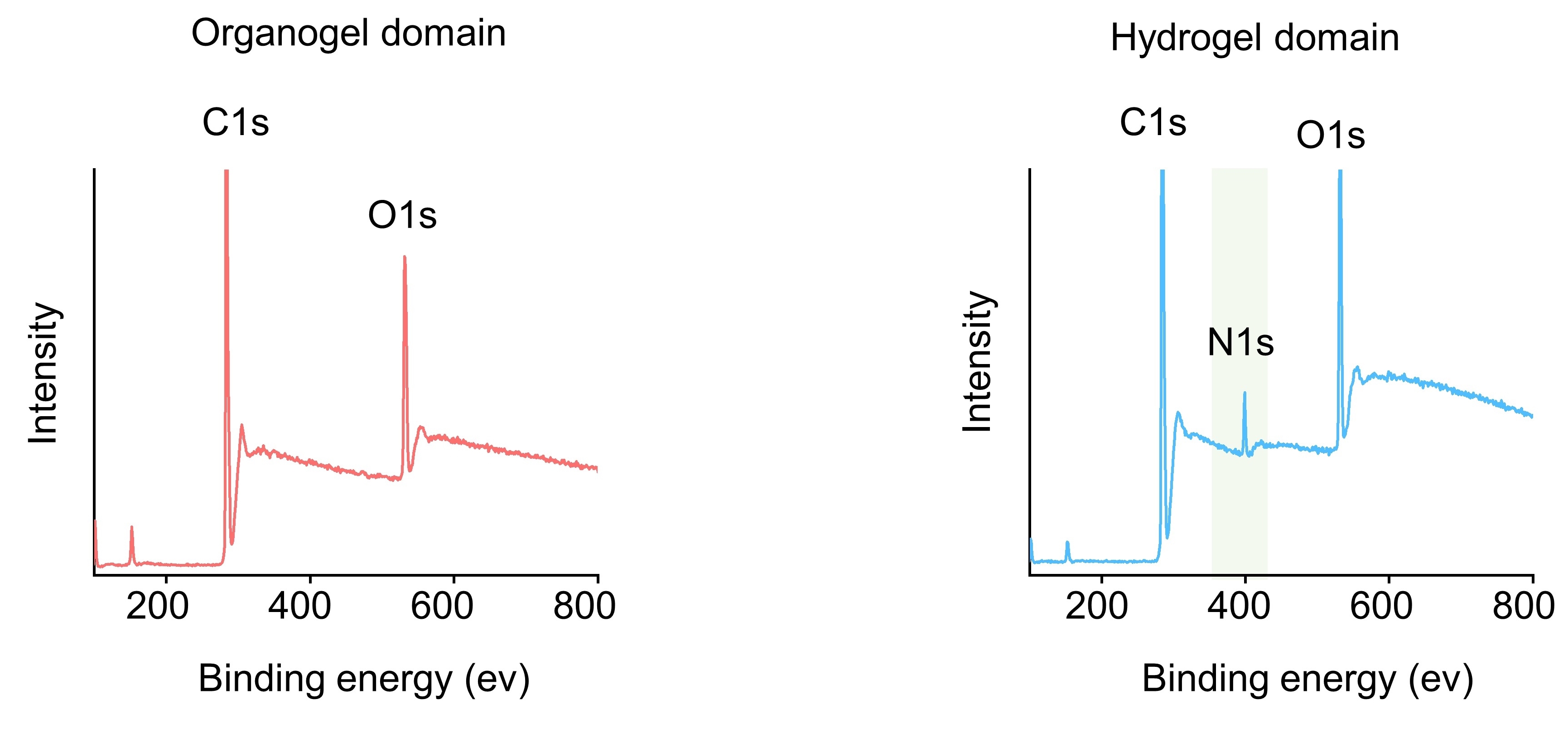


**Figure S1.** The XPS analysis confirmed the composition of the hydrogel and organogel domains of the top surface, respectively.


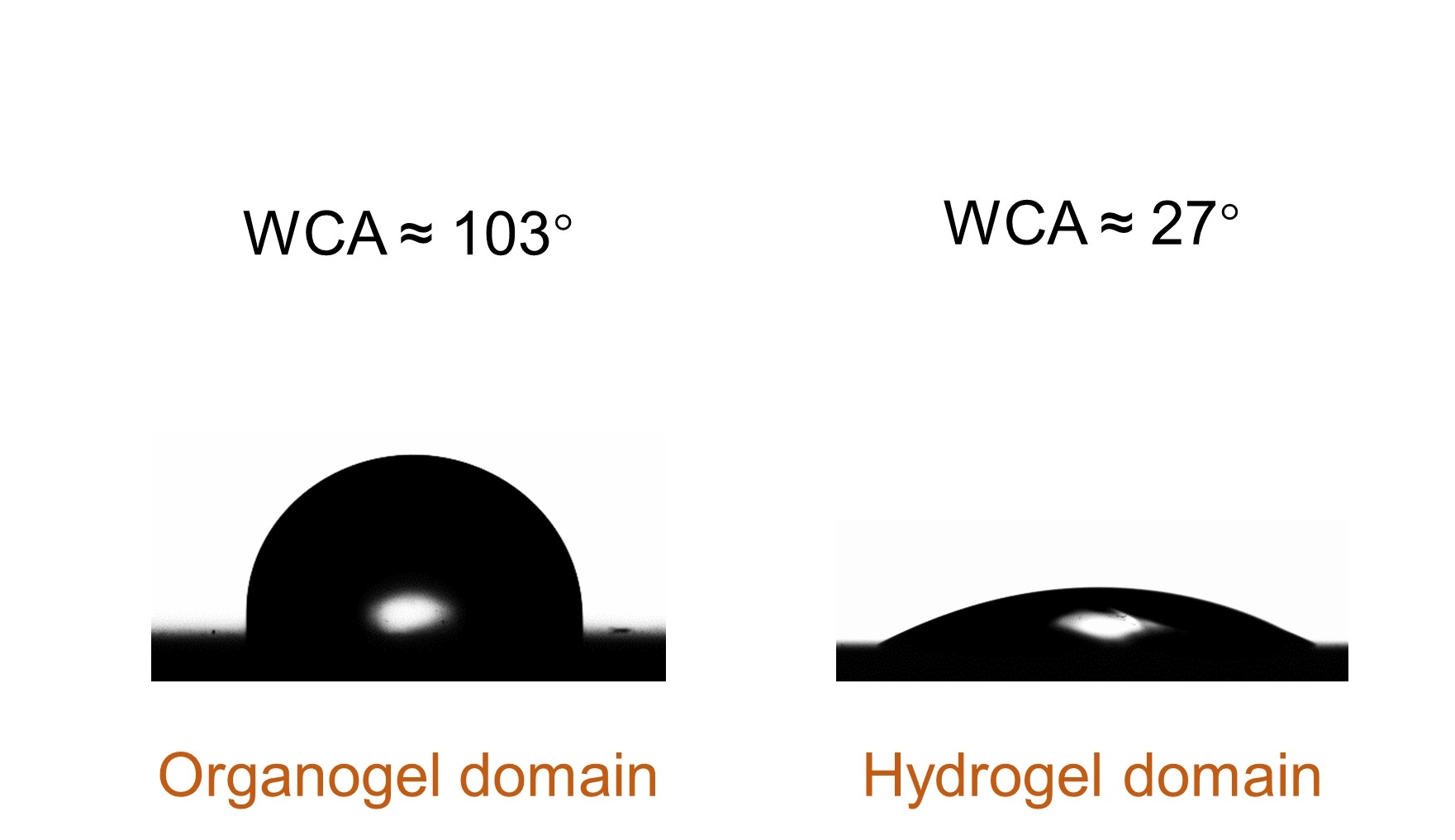


**Figure S2.** The water contact angle of the organogel domain and hydrogel domain on the top surface.


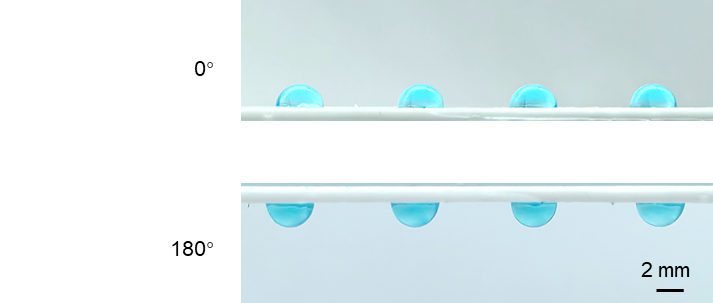


**Figure S3.** The ability of the heterogeneous organohydrogel to anchor the droplet array. Capture of blue-dyed droplet array with different rotation angles (0° and 180°).


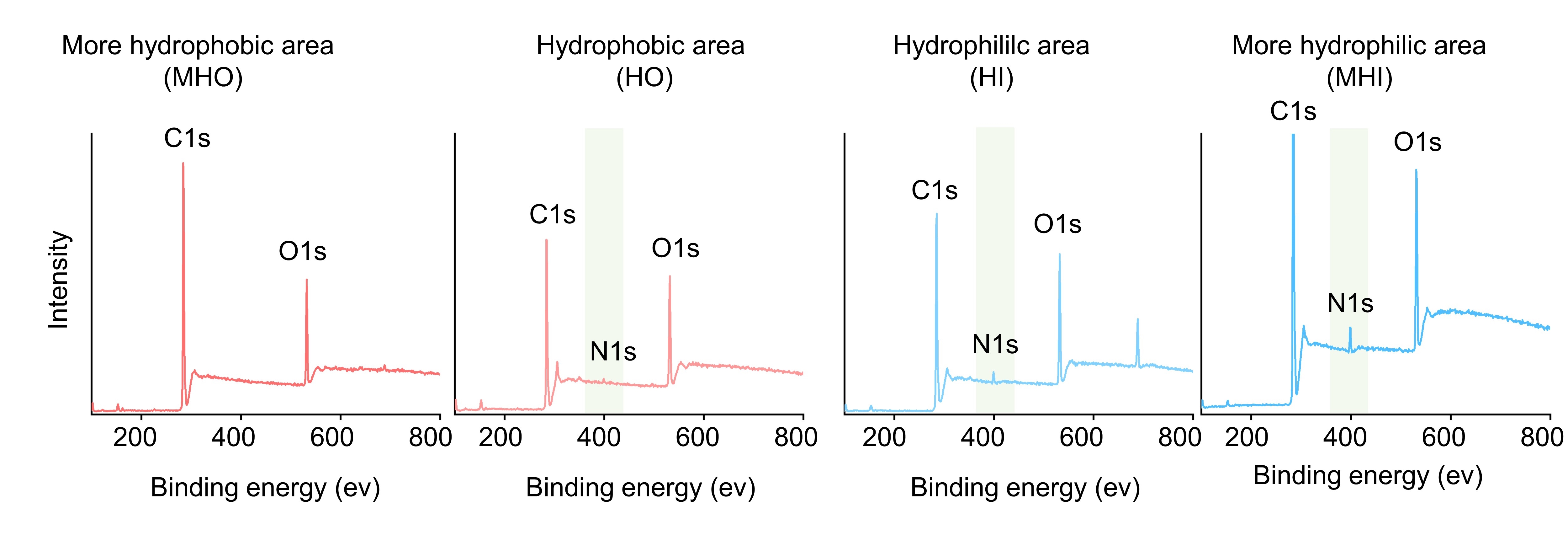


**Figure S4.** The XPS analysis confirmed the composition of the different regions of the bottom surface.


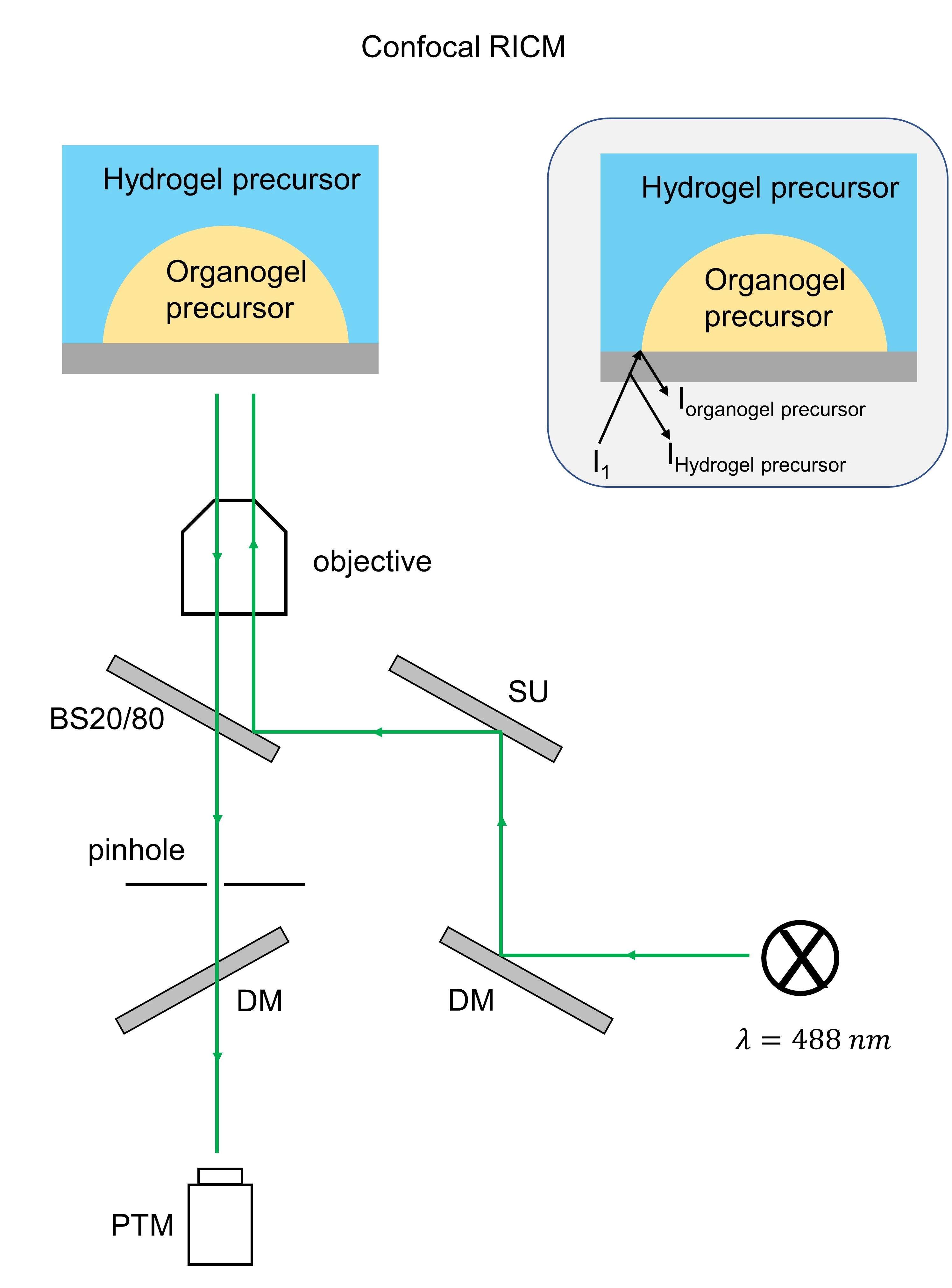


**Figure S5.** Schematic of the RICM set-up used to visualize the intercalated oil film formed by organogel precursor. DM: dichroic mirror, SU: galvanometric mirror (GM), BS20/80: semi-reflective mirror (SRM), PMT: photomultiplier tube.


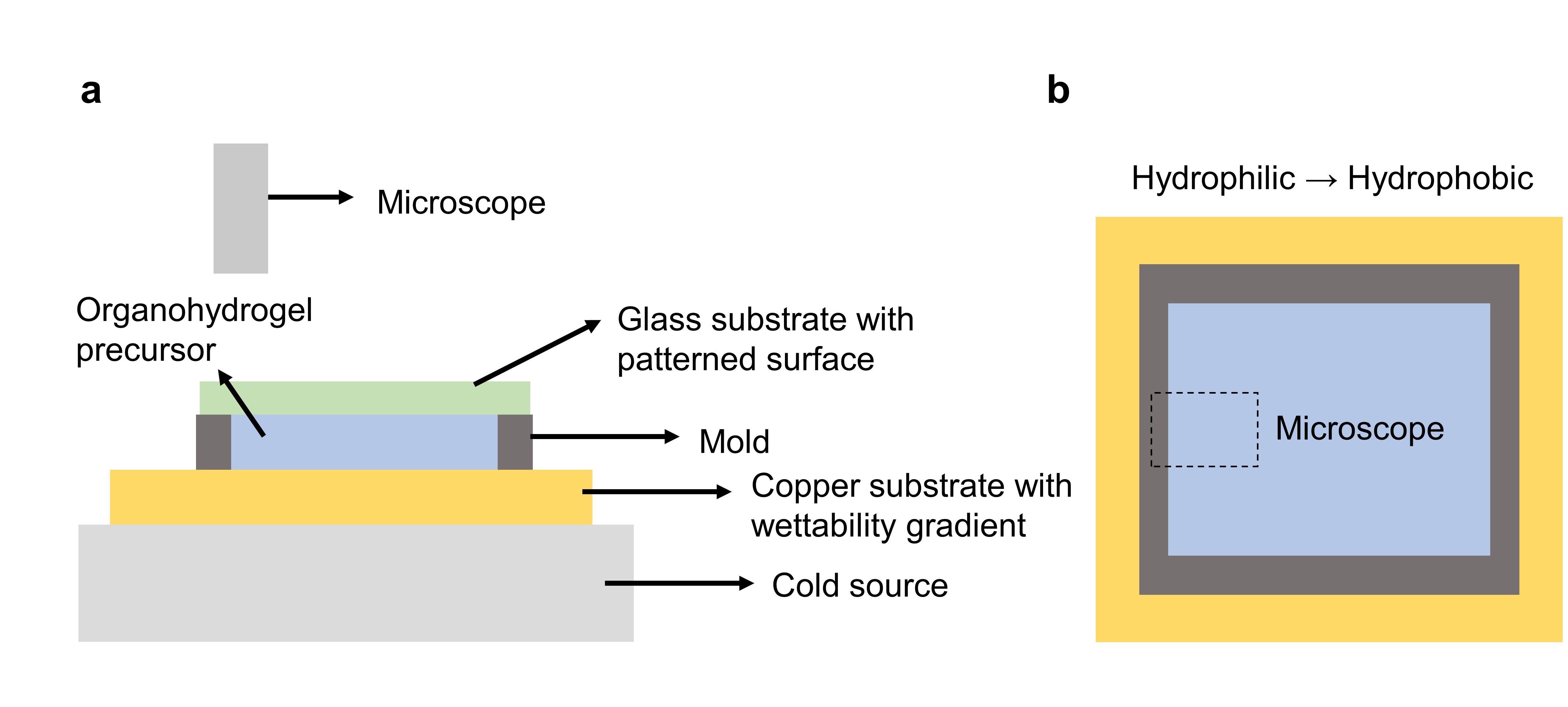


**Figure S6.** Setup for observing the freezing process. A) A mold (80 × 80× 1 mm) was sealed to the copper substrate with gradient wettability. During observation, the setup was put on a cold source. B) The freezing process was observed in the top view at the most hydrophilic region.


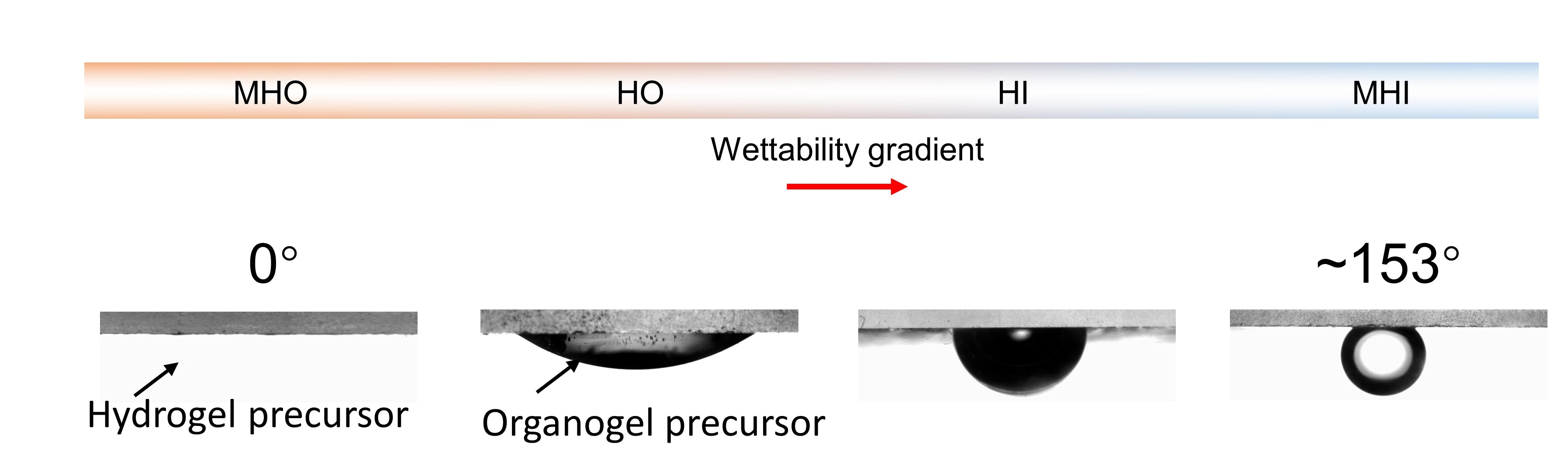


**Figure S7.** The underwater oil contact angle of the droplets of organogel precursor on each region of the wettability-gradient substrate.


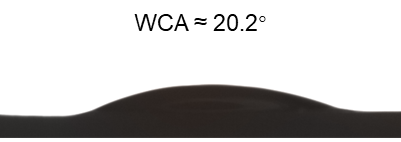


**Figure S8.** The WCA of organohydrogel bulk.


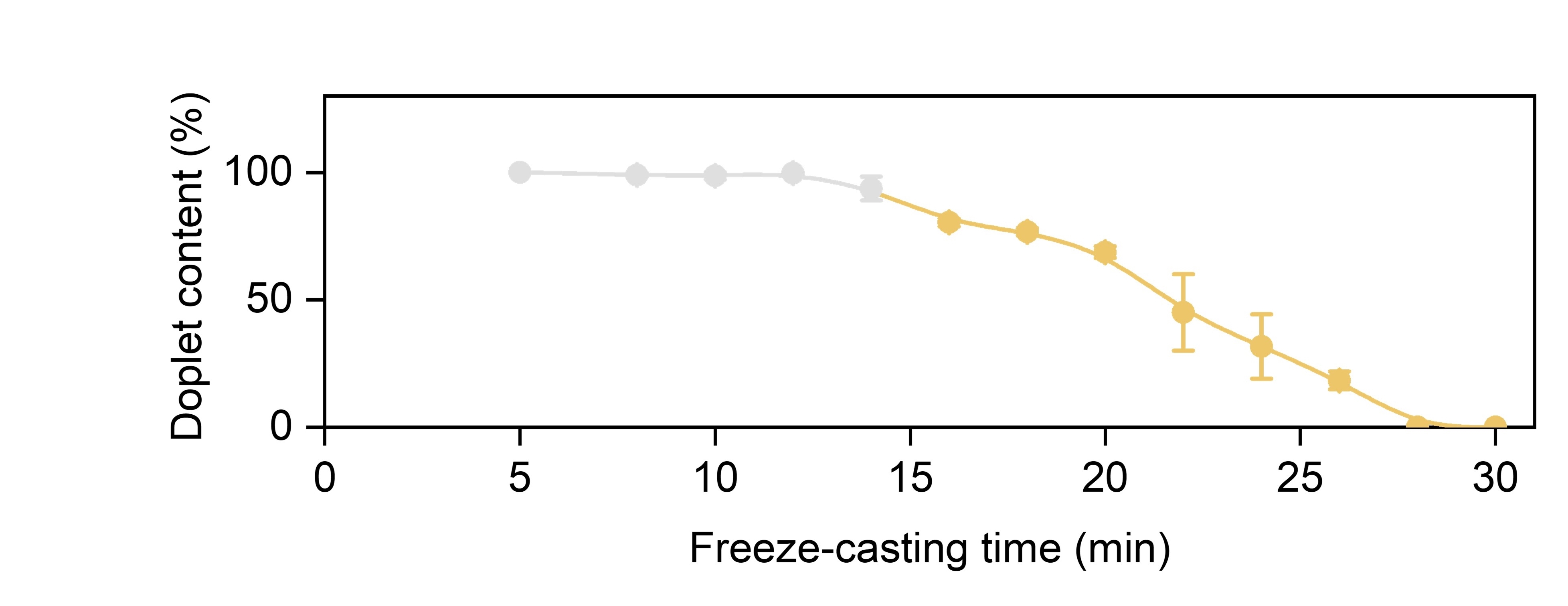


**Figure S9.** The water content of droplets dripped on the MHO area of the bottom surface as a function of freeze-casting time.


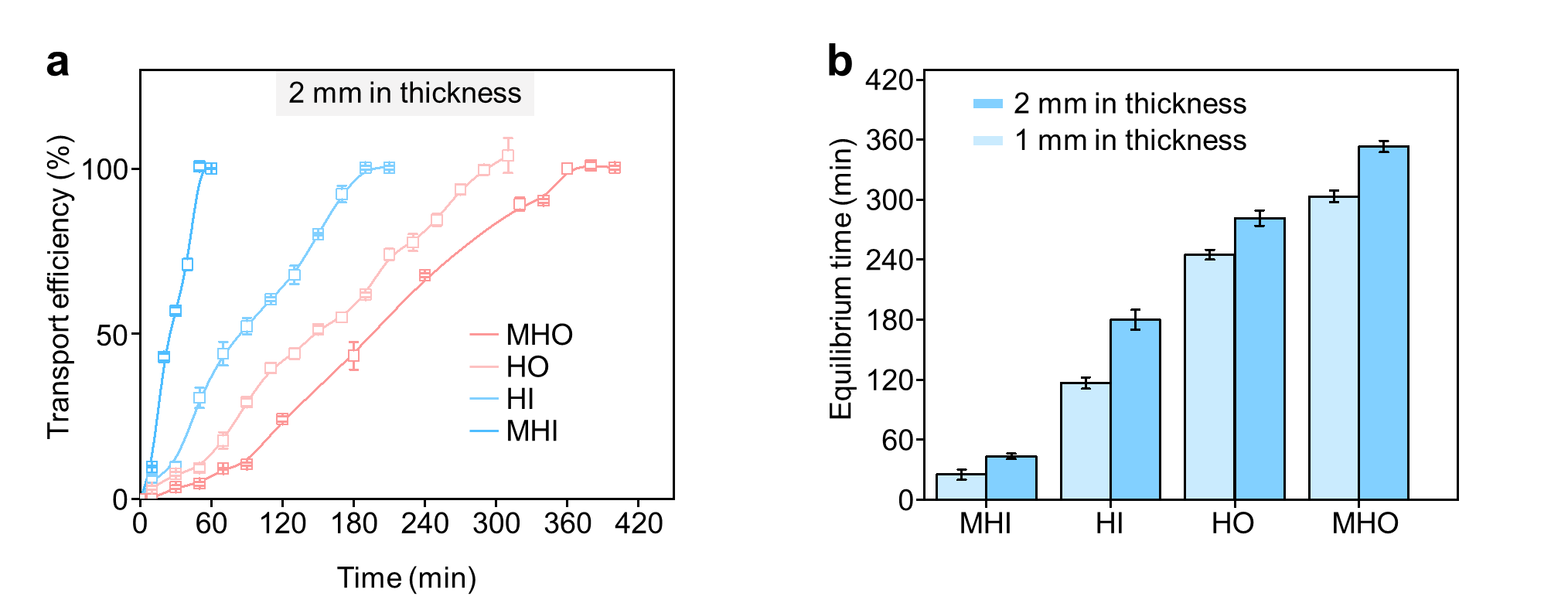


**Figure S10.** The effect of the thickness of heterogeneous organohydrogels on transport efficiency. a) The cumulative release profiles of sodium fluorescein from the bottom surface to the droplet array on the top surface using the noncontact method (2 mm in thickness, 1 mm in diameter of hydrogel region). b) Comparison of equilibrium time for heterogeneous organohydrogels with different thicknesses.


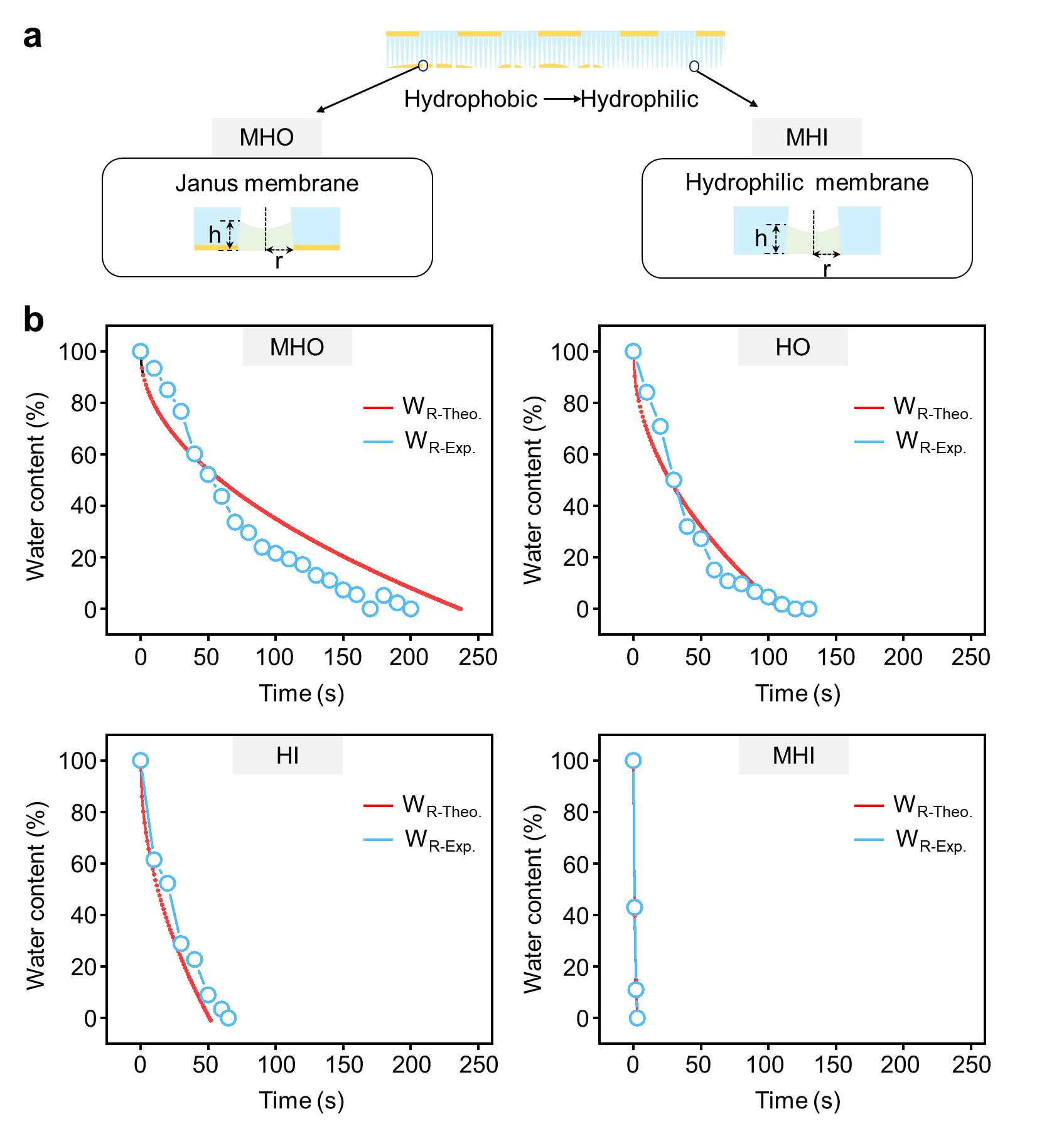


**Figure S11.** Capillary rise dynamics of droplet through the heterogeneous organohydrogel. a) Schematic of capillary microstructure of MHO and MHI regions. b) Comparison of experimental residual water content (blue lines) and theoretical elevation residual water content (red lines) on different regions of heterogeneous organohydrogel bottom surface as a function of time.


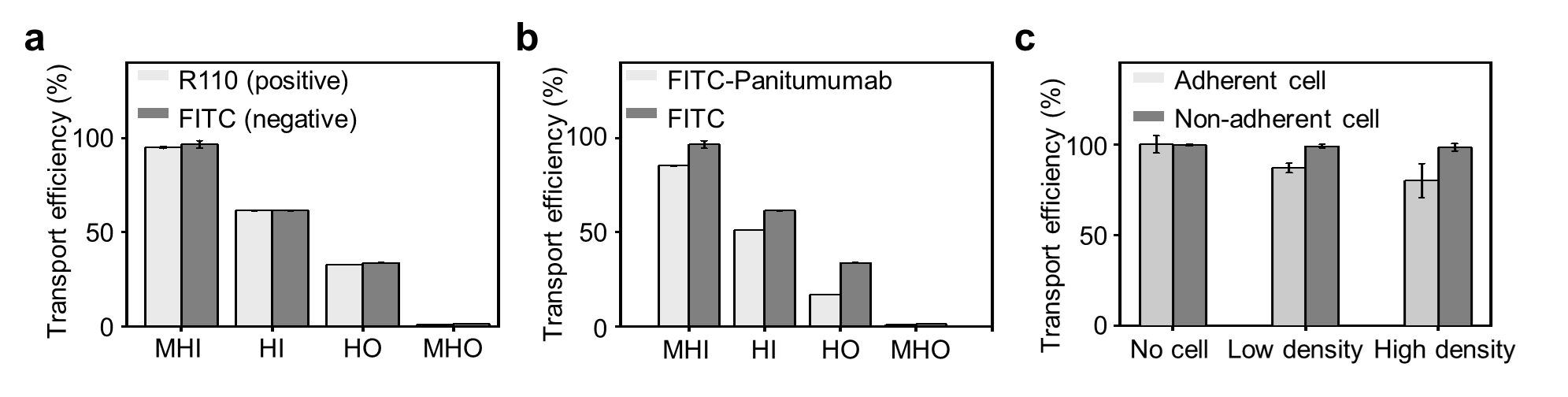


**Figure S12**. The effect of charge, molecular weight of drug and cell density on transport efficiency of the heterogeneous organohydrogel. Transport time was set to 20 min. a) Due to the very low electronegativity of the heterogeneous organohydrogel (-1.36 ± 1.34 mV), the transport efficiency is similar for drugs with different charged properties. b) When transporting macromolecules such as FITC-panitumumab (Mw=143 kDa), the transport efficiency was slightly reduced. c) For non-adherent cells (Jurkat), the transport efficiency at higher cell densities (1.2 × 10^5^ cells mL^−1^) is consistent with that at lower cell densities (1.2 × 10^4^ cells mL^−1^) and in the absence of cells. For adherent cells (PC3), as the cell density increases from 3.0 × 10^4^ cells mL^−1^ to 3.0 × 10^5^ cells mL^−1^, the transport efficiency gradually decreases from ~100% to ~80%, which may be due to the adherent cells inhibiting the drug diffusion.

**
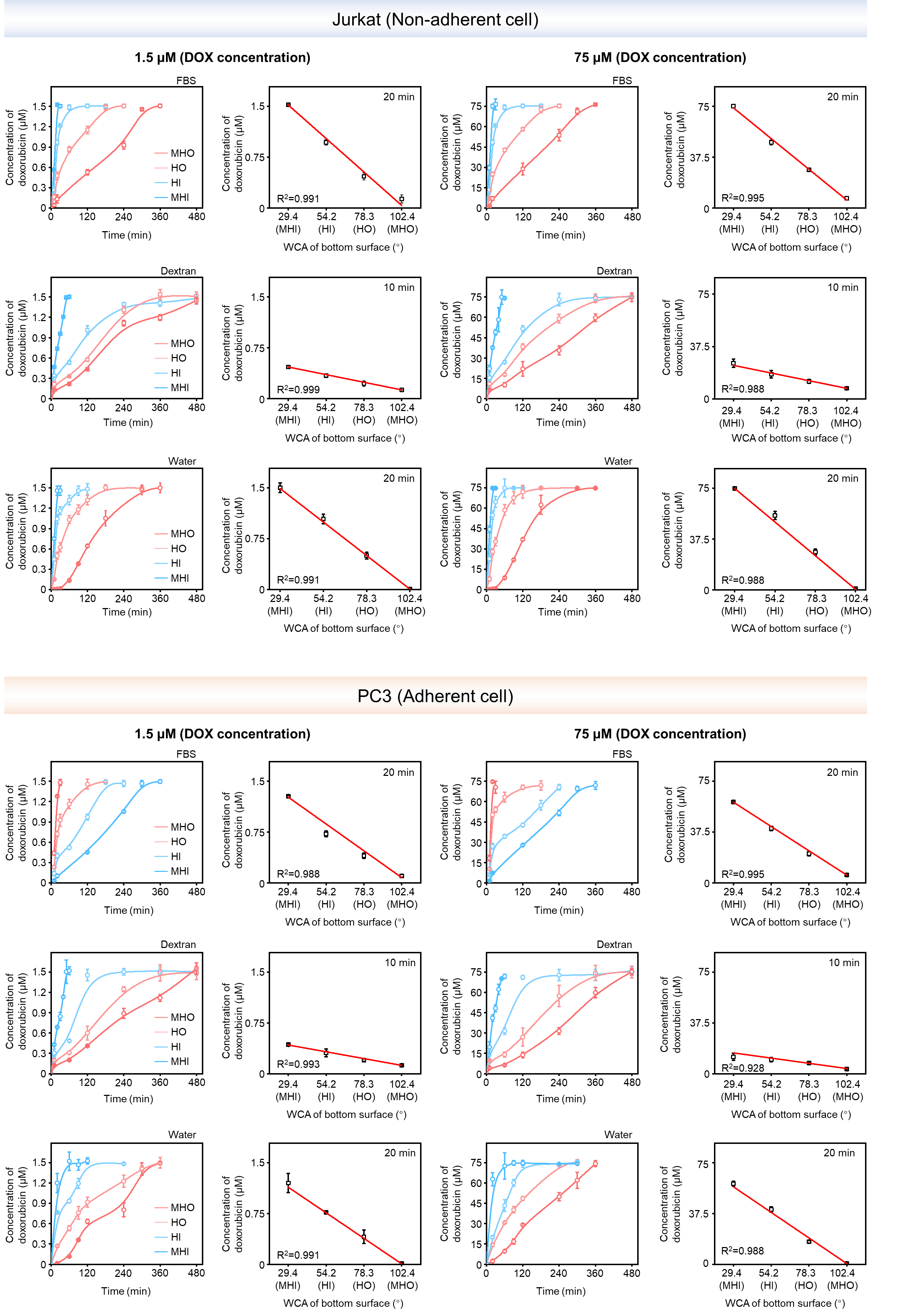
**

**Figure S13**. Relationship of water contact angle of bottom surface and gradients in different solvents and at different providing concentrations. The signal was determined from the fluorescence signal of doxorubicin. The transported doxorubicin is proportional to the water contact angle of the bottom surface.


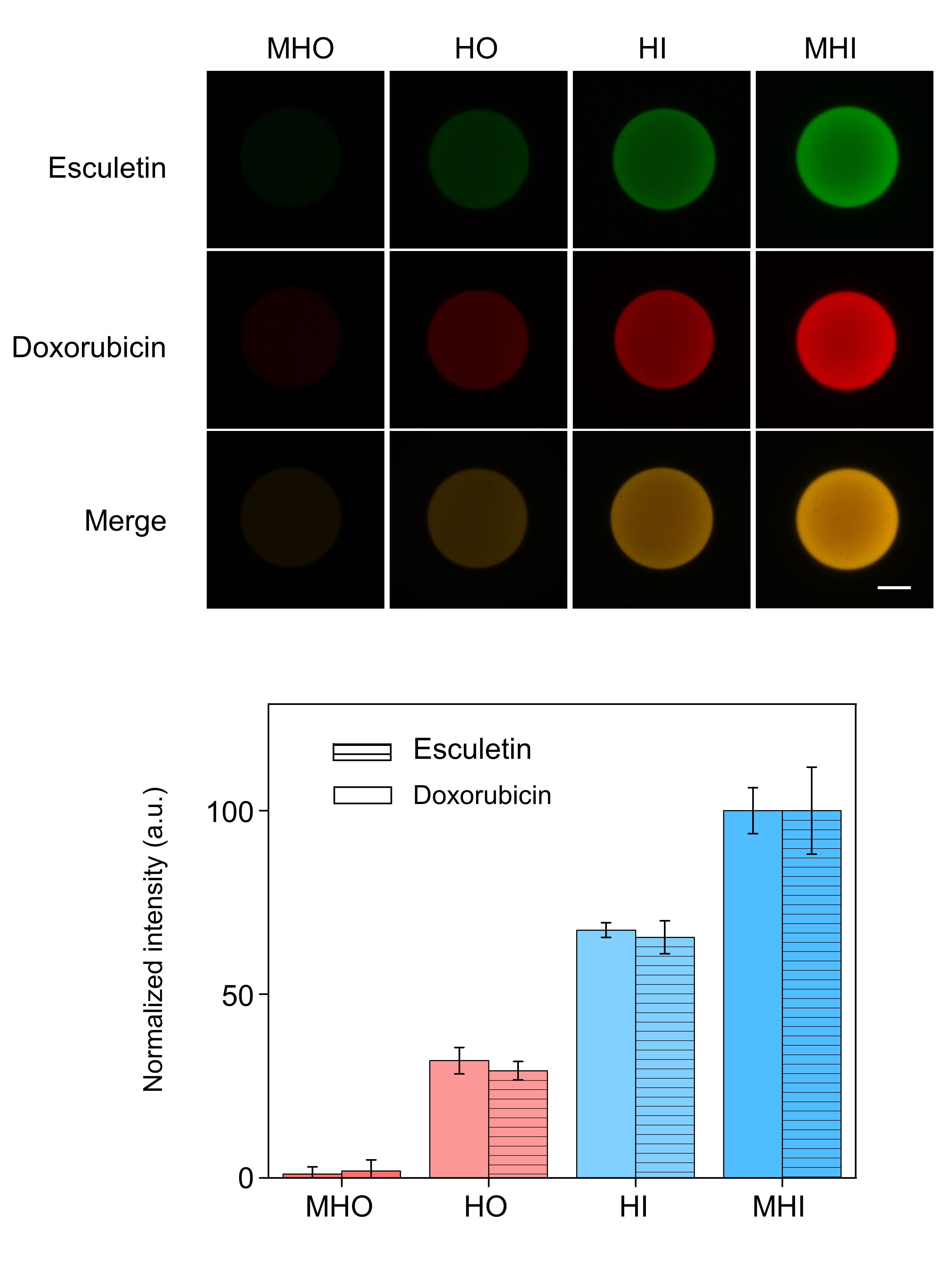


**Figure S14.** The fluorescence images and quantification of total released contents showed a droplet array on the heterogeneous organohydrogel with bio-component gradients in esculetin and doxorubicin from the MHO to the MHI region. The excitation wavelengths were 405 nm and 488 nm for esculetin and doxorubicin, respectively. Scale bar: 200 μm.


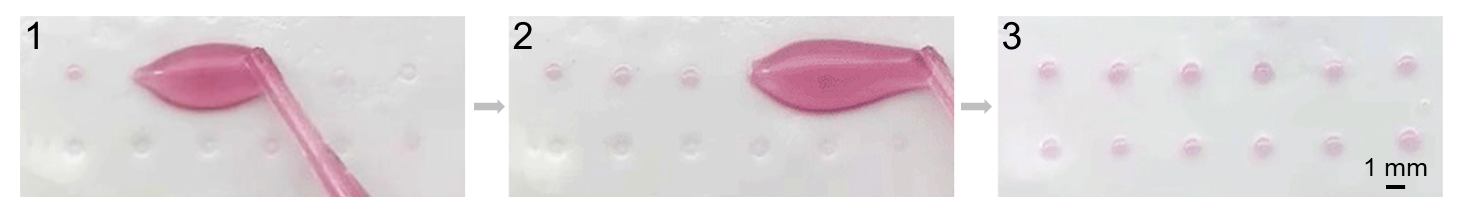


**Figure S15.** Snapshots of culture medium containing cells being moved along the heterogeneous organohydrogel (1 mm diameter hydrogel domain) to form an array of cell-based microdroplets by discontinuous dewetting.


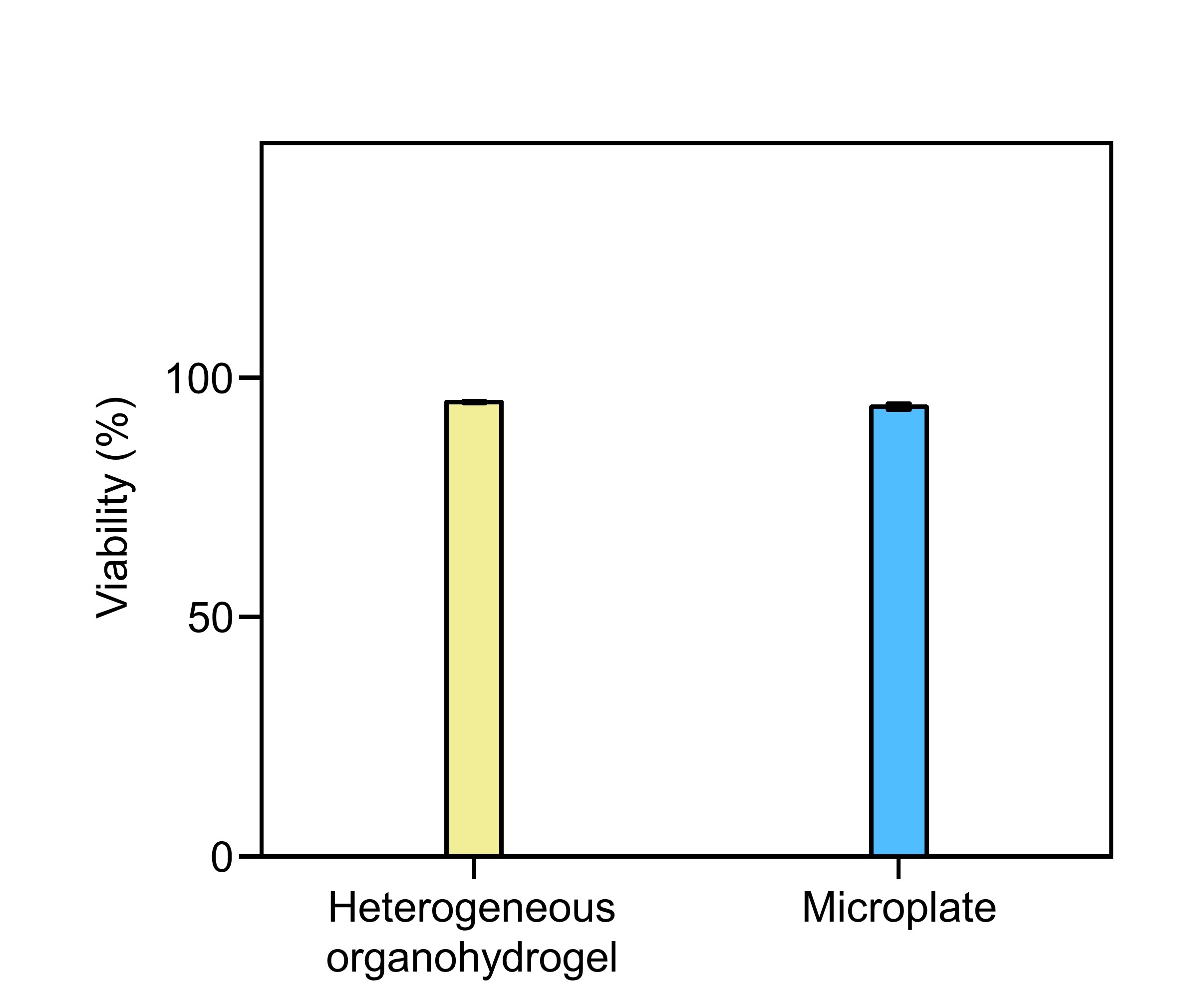


**Figure S16.** Viability of Jurkat cells culture on the top surface of the heterogeneous organohydrogel and a 96-well microplate.


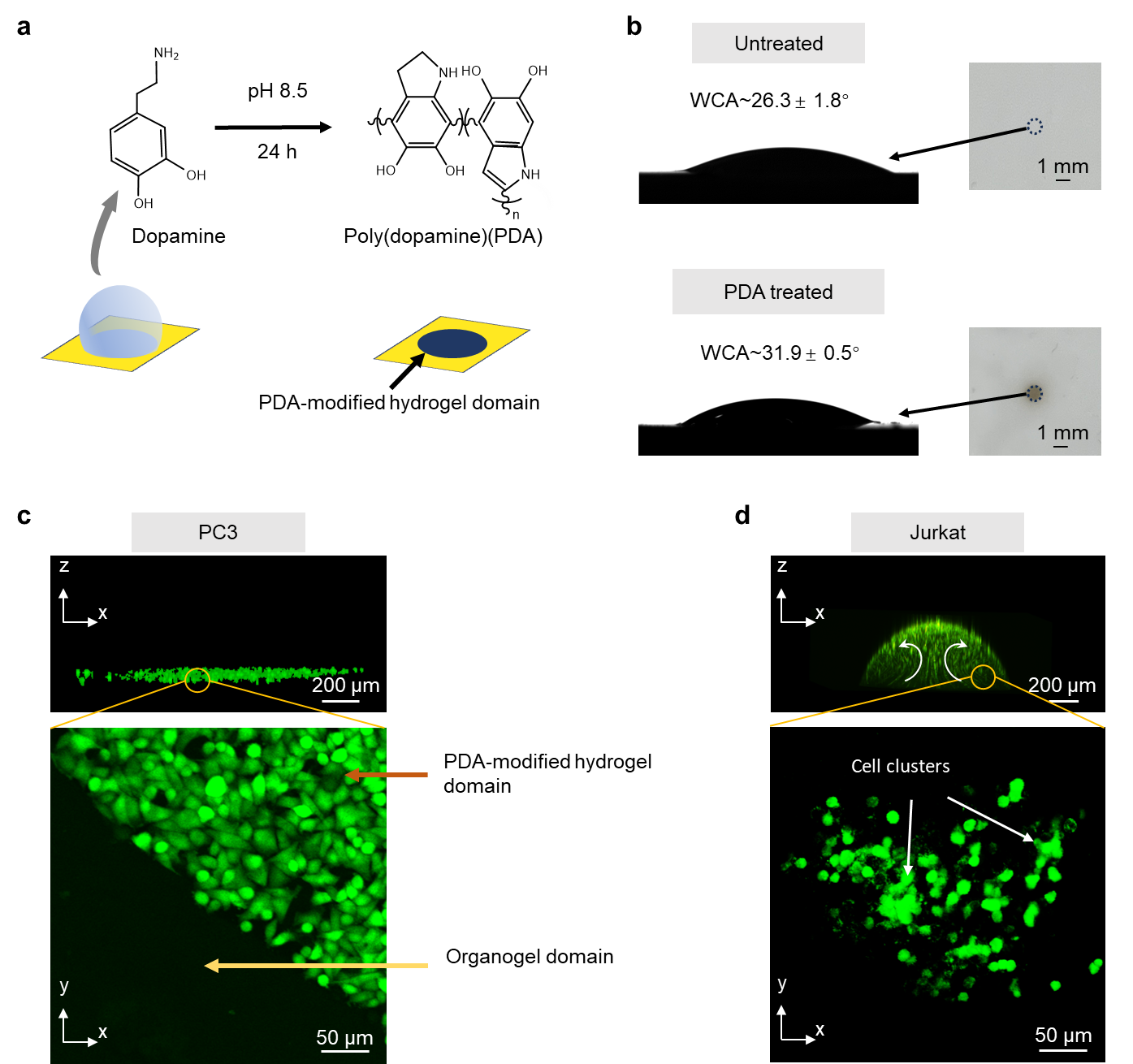


**Figure S17.** PC3 and Jurkat cells cultured on the top surface of the heterogeneous organohydrogel. a) Surface PDA modification of heterogeneous organohydrogel is achieved simply by incubating the hydrogel domain with a dopamine hydrochloride solution to form a thin surface coating. b) The PDA-functionalized hydrogel domain shows an increased water contact angle as compared to the untreated hydrogel domain. After 24 hours, PDA was prepared on the hydrogel domain and therefore the color of the hydrogel domain changed to black-brown. Cross-sectional and top-view CLSM images showing the distribution and structure of PC3 cells (c) and Jurkat cells (d) on the hydrogel domain of the top surface.


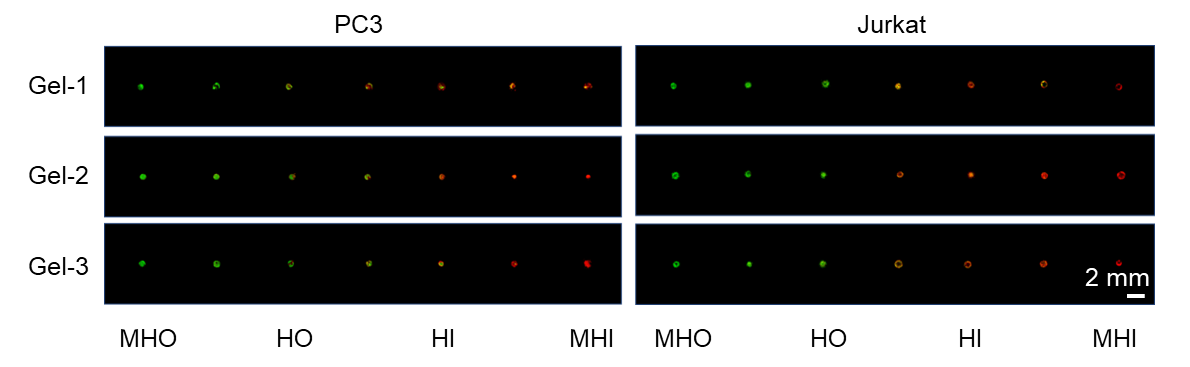


**Figure S18.** CLSM images of PC3 and Jurkat cells on the heterogeneous organohydrogels after doxorubicin screening. The doxorubicin used for PC3 and Jurkat cells was 1.5 μM and 75 μM, respectively.


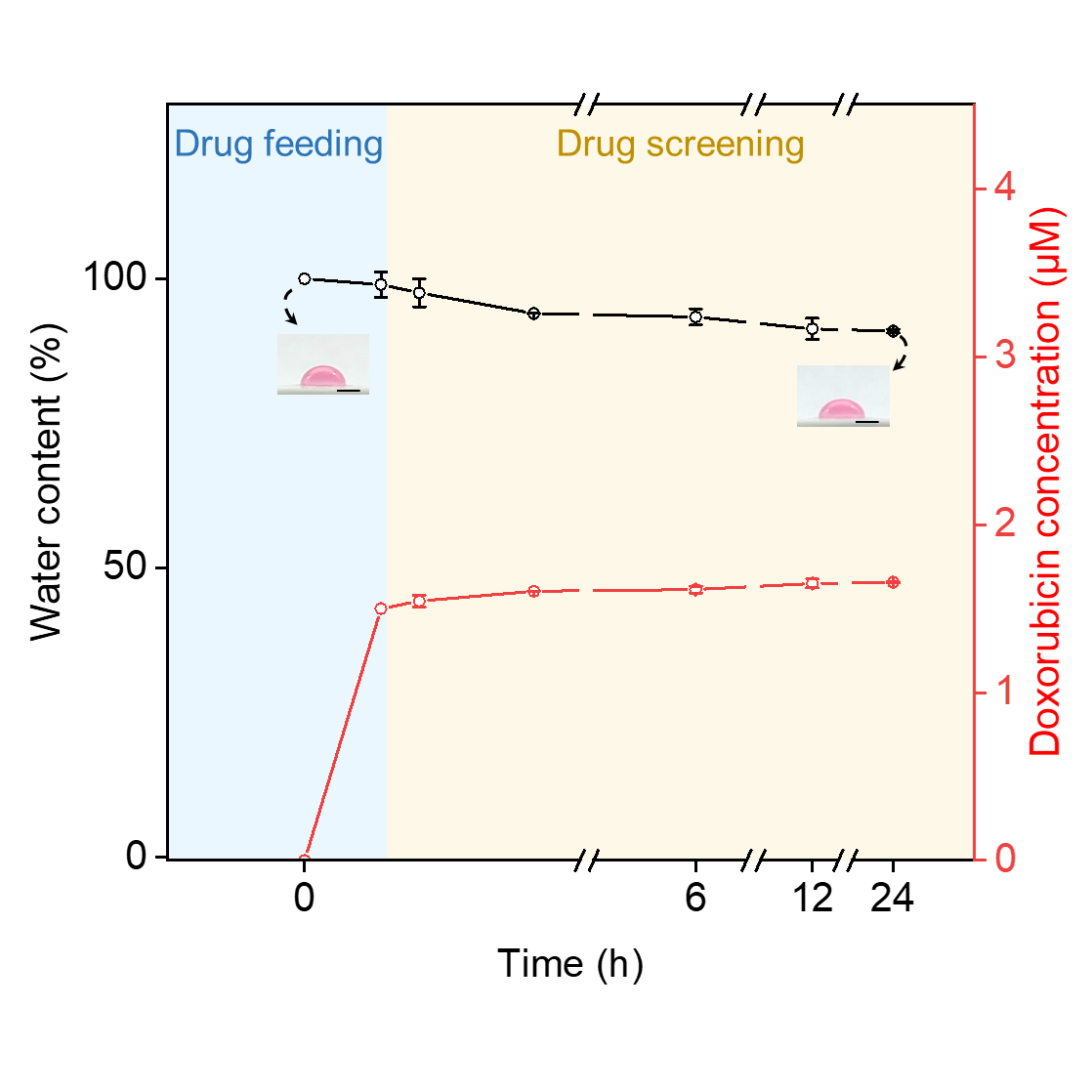


**Figure S19.** The variation of droplet content and concentration of doxorubicin (1.5 μM) in droplets within 24 h during drug screening through noncontact method. Scale bar, 1 mm.


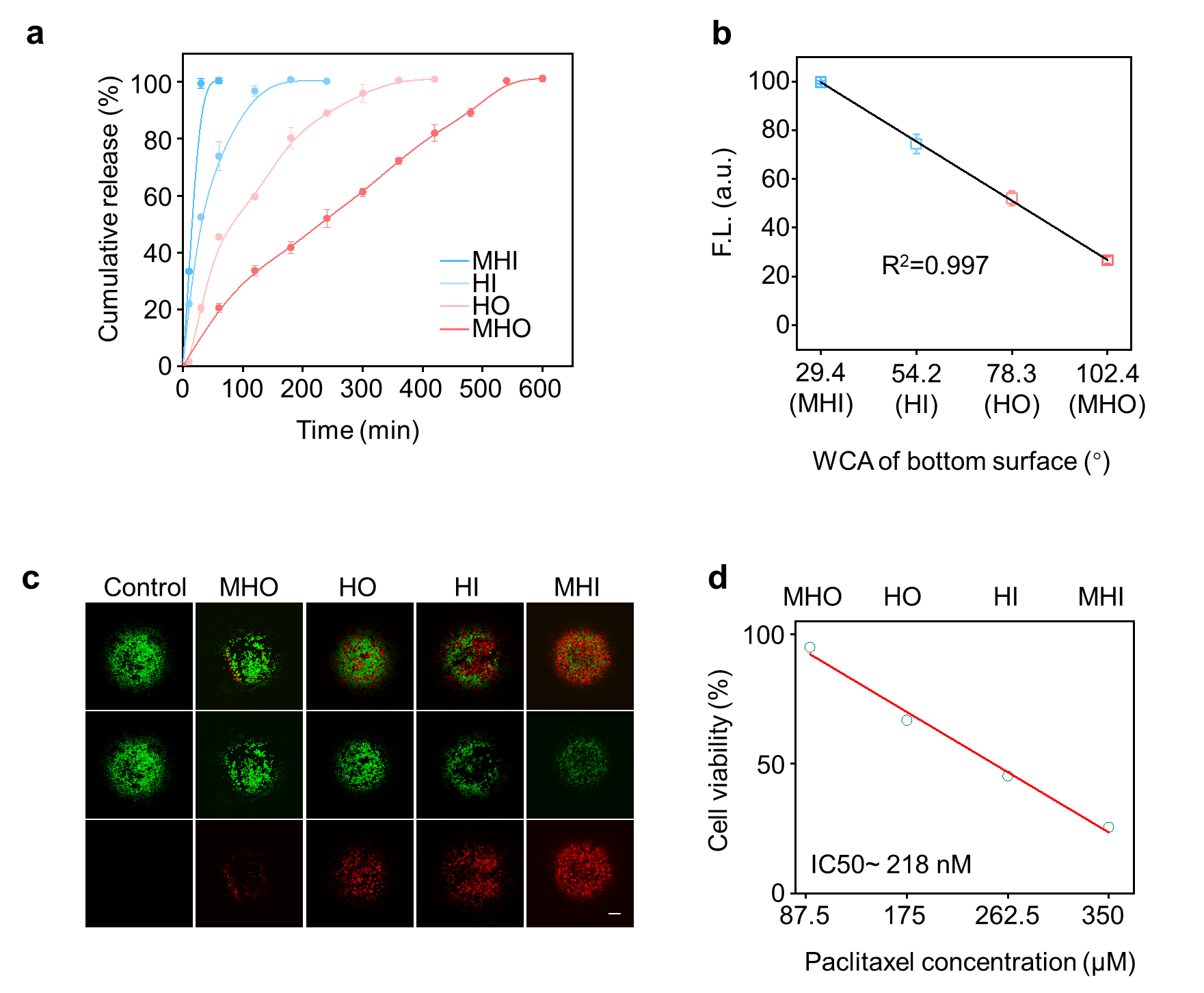


**Figure S20.** Gradient feeding of paclitaxel dissolved in DMSO to the cell-based array on the top surface of heterogeneous organohydrogel using a noncontact method. a) The quantitative analysis of the cumulative release of cy5-paclitaxel from the bottom surface to the droplet array on the top surface. b) The fluorescent intensity and contact angles of the bottom surface are linearly related. c) Microscope images of Jurkat cells cultured in the heterogeneous organohydrogel treated with paclitaxel and (d) the concentration-dependent effect of paclitaxel on the viability of Jurkat cells 24 h after treatment. Scale bar: 200 μm.

**Table S1.** The influence of the selected hydrogel and organogel monomers on the WCA range of the wettability-gradient surface. AM: acrylamide, AA: acrylic acid, HEMA: hydroxyethyl methylacrylate, SBMA: 2-(Methacryloyloxy)ethyl]dimethyl-(3-sulfopropyl), BMA: butylmethacrylate, PEGDA: poly (ethylene glycol) diacrylate.


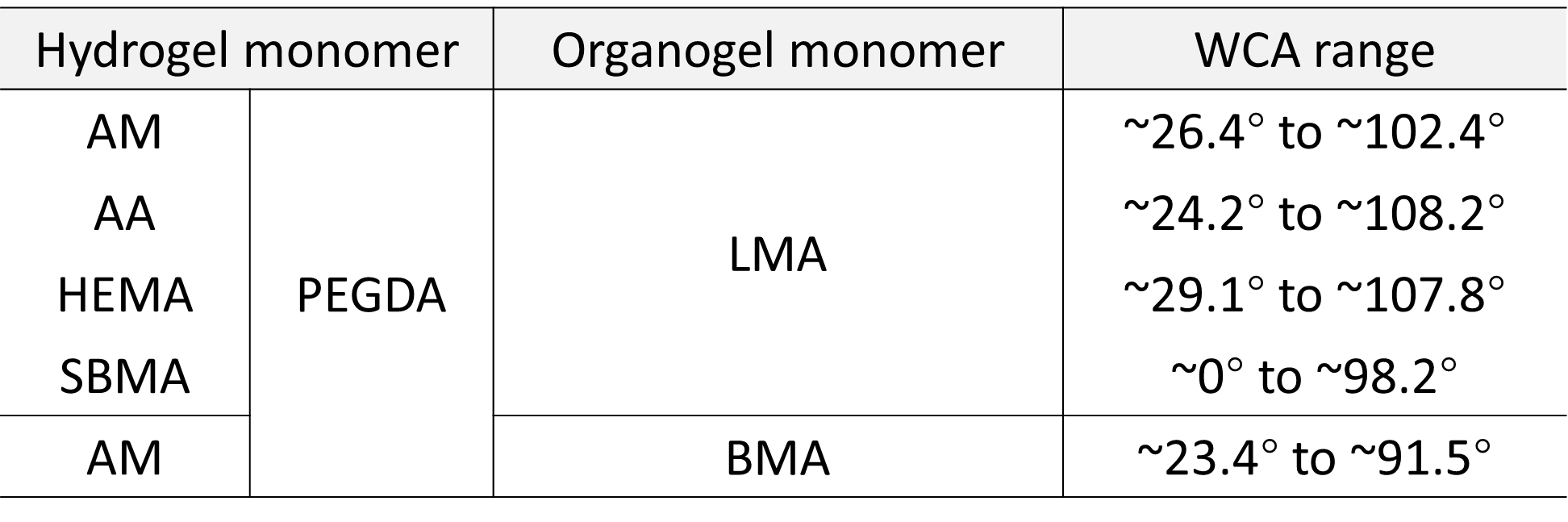


**Table S2.** The competition of organogel precursors and hydrogel precursors on different regions of wettability-gradient surface (Note: In our oil-in-water emulsion system, liquid A (hydrogel precursor) is the continuous phase, liquid B (organogel precursors) is the dispersed phase. “N” indicates that liquid B cannot displace liquid A; “Y” indicates that liquid B can displace liquid A. “P” indicates that liquid B can displace liquid A partially. All the data were repeated more than three times.


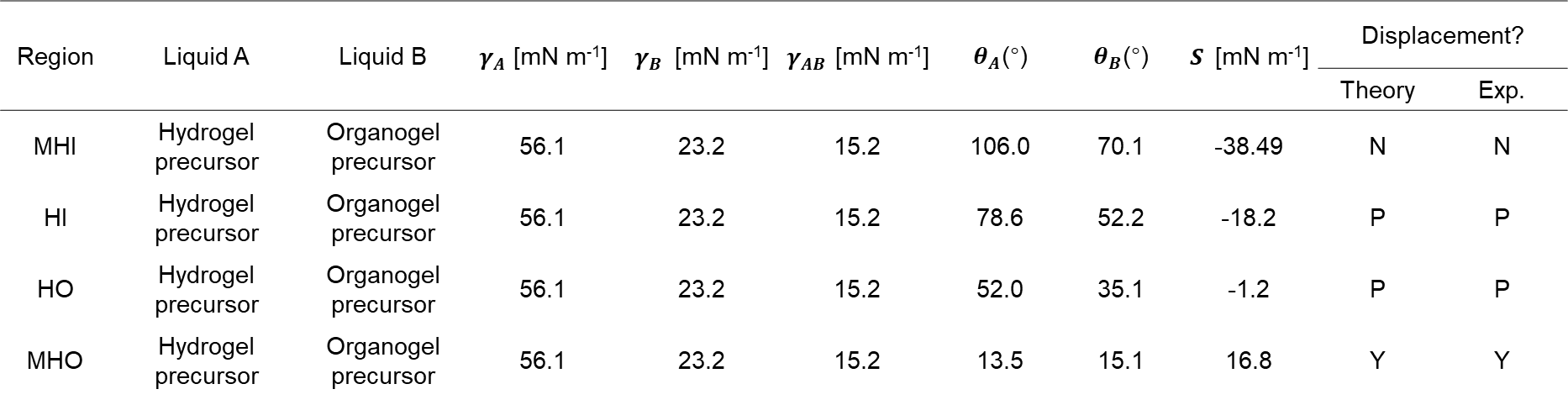


**Table S3.** Dosing time table for screening of adherent and non-adherent cells using doxorubicin (DOX) dissolved in different solvents.


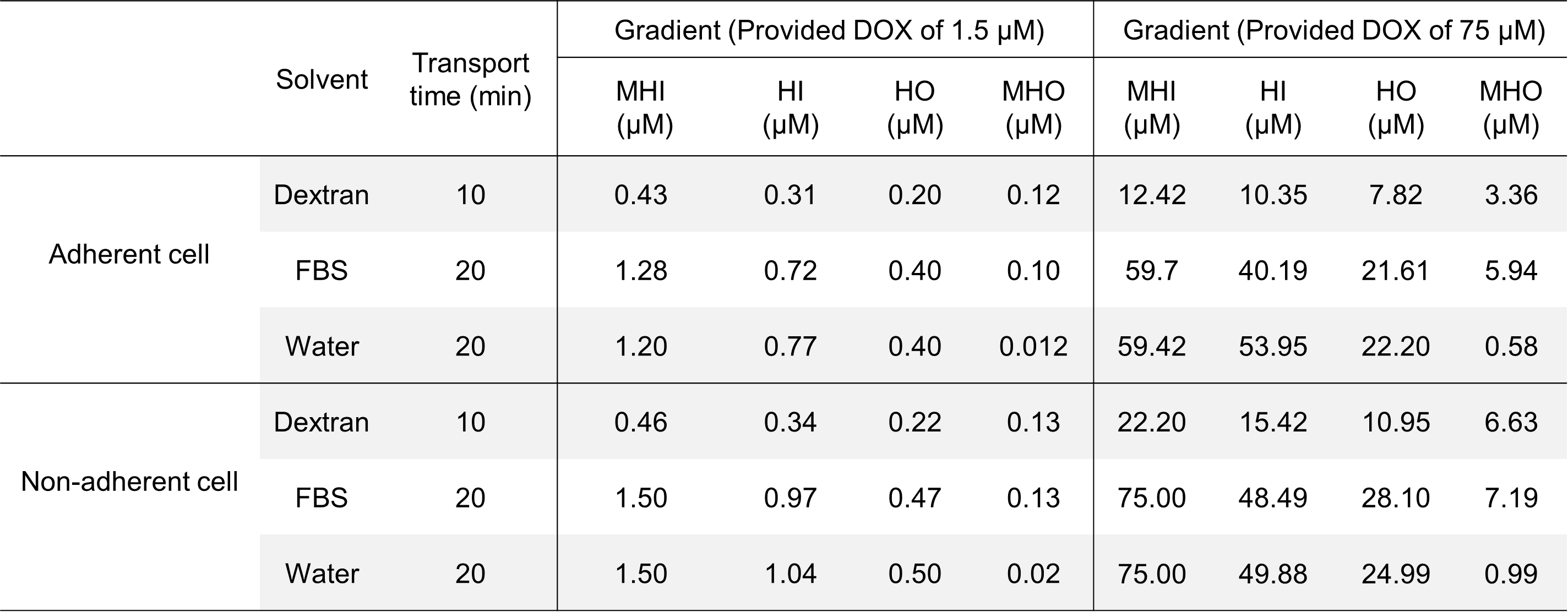


**Table S4.** Comparison of IC_50_ of doxorubicin on PC3 and Jurkat cells via different methods.


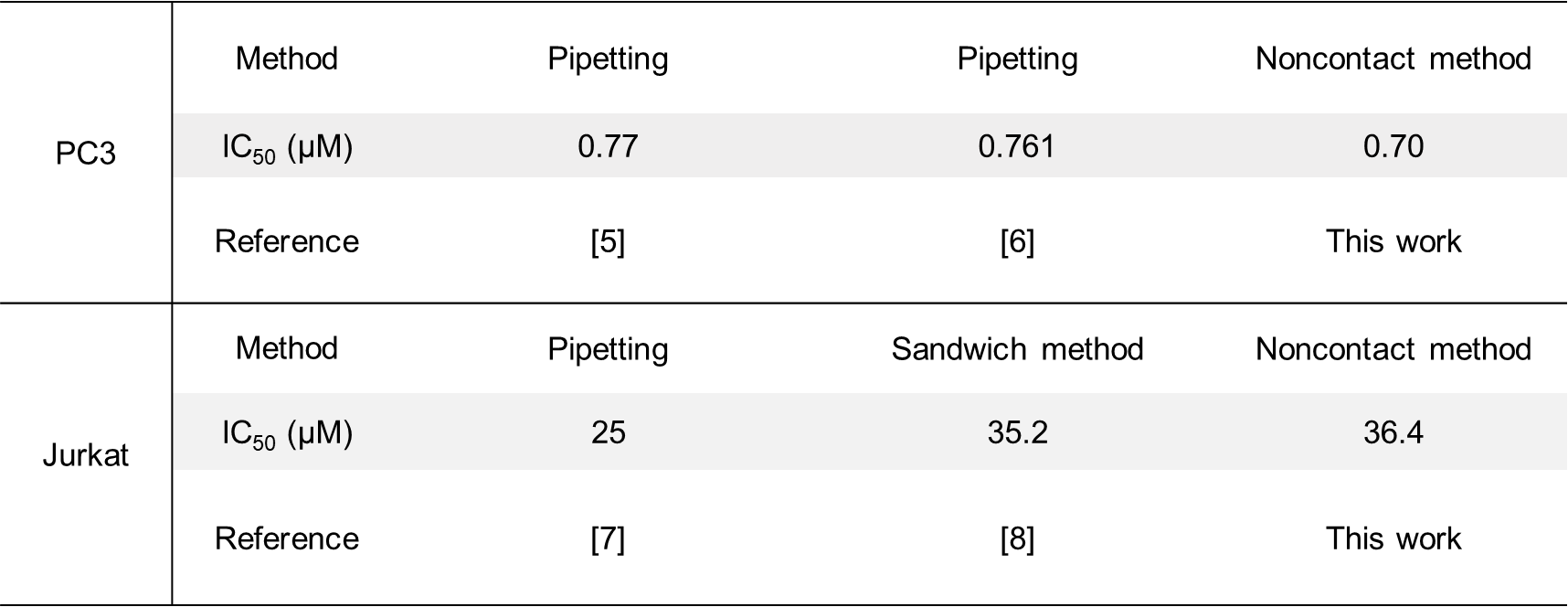


**Movie S1.** The process of the VB_1_ molecule (labeled red) is transported at a gradient velocity to the droplet array on the top surface of the heterogeneous organohydrogel. The VB_1_ molecule reacted with the colorimetric reagent in the droplet array to produce the orange-red product.

Red product

**3. Supplementary References**

[1] S. Li, A. Sng, D. Daniel, H. C. Lau, O. Torsæter, L. P. Stubbs, *ACS Appl. Mater. Interfaces* **2021**, 13, 41182-41189.

[2] A. Siebold, A. Walliser, M. Nardin, M. Oppliger, J. Schultz, *Journal of Colloid and Interface Science* **1997**,186(1), 60-70.

[3] S. Daniel, M. K. Chaudhury, J. C. Chen, *Science* **2001**, 291, 633-636.

[4] M. K. Chaudhury, G. M. Whitesides, *Science* **1992**, 256, 1539-1541.

[5] D. C. Wu, C. R. Cammarata, H. Joo Park, B. T. Rhodes, C. M. Ofner III, *Pharm. Res.* **2013**, 30, 2087-2096.

[6] C. H. Trebelhorn, J. C. Dennis, S. R. Pondugula. T. Samuel, E. Coleman, P. Flannery, E. Morrison, M. Mansour, *J. Cancer Res. Ther*. **2014**, 2(9), 132-143.

[7] D. Ivanova, Z. Zhelev, S. Semkova, I. Aoki and R. Bakalova, *Anticancer Research* **2019**, 39(7), 3745-3755.

[8] A. A. Popova, S. Dietrich, W. Huber, M. Reischl, R. Peravali, P. A. Levkin, *SLAS Technology* **2021**, 26(3), 274-286.
